# Spatio-temporal neural stem cell behavior leads to both perfect and imperfect structural brain regeneration in adult newts

**DOI:** 10.1242/bio.033142

**Published:** 2018-06-14

**Authors:** Yuko Urata, Wataru Yamashita, Takeshi Inoue, Kiyokazu Agata

**Affiliations:** 1 Department of Biophysics, Graduate School of Science, Kyoto University, Kyoto, 606-8502, Japan; 2 Developmental Neurobiology, Kyoto Prefectural University of Medicine, Kyoto, 606-0823, Japan; 3 Department of Life Science, Gakushuin University, Tokyo, 171-8588, Japan

**Keywords:** Regeneration, Central nervous system, Neurogenesis, Neural stem cells

## Abstract

Adult newts can regenerate large parts of their brain from adult neural stem cells (NSCs), but how adult NSCs reorganize brain structures during regeneration remains unclear. In development, elaborate brain structures are produced under broadly coordinated regulations of embryonic NSCs in the neural tube, whereas brain regeneration entails exquisite control of the re-establishment of certain brain parts, suggesting that a yet-unknown mechanism directs NSCs upon partial brain excision. Here we report that upon excision of a quarter of the adult newt (*Pleurodeles waltl*) mesencephalon, active participation of local NSCs around specific brain subregions’ boundaries leads to some imperfect and some perfect brain regeneration along an individual's rostrocaudal axis. Regeneration phenotypes depend on how wound closing occurs using local NSCs, and perfect regeneration replicates development-like processes, but takes more than 1 year. Our findings indicate that newt brain regeneration is supported by modularity of boundary-domain NSCs with self-organizing ability in neighboring fields.

This article has an associated First Person interview with the first author of the paper.

## Abstract (precis)

**Summary:** Upon large-scale brain excision in adult newts, the outcome of structural regeneration of the brain cytoarchitecture depends on ways of closing the wound by NSCs residing between brain subregions.

## INTRODUCTION

Adult urodele amphibians and teleosts have a remarkable ability to regenerate large parts of their brain and spinal cord, an ability that is based on the broad distribution of adult neural stem cells (NSCs) throughout the ventricular zone of their central nervous system (CNS) (Alunni and Bally-Cuif, 2016; Kaslin et al., 2008; Tanaka and Ferretti, 2009). Although adult mammals also maintain NSCs in neurogenic niches in their brain, and numerous attempts have been made to elicit the regenerative response in adult mammalian NSCs as a therapeutic target (Kolb et al., 2007; Schänzer et al., 2004; Thau-Zuchman et al., 2010), large-scale brain regeneration remains a formidable challenge (Obernier et al., 2014).

In contrast to mammals, adult NSCs in urodele amphibians and teleosts, referred to as ‘ependymoglia cells’, act as a source of cells for both CNS regeneration and tissue homeostasis (Becker and Becker, 2015). Ependymoglia cells lining the apical surface of the brain are tightly connected to each other to form the ependymal layer, and typically possess a long radial process extending toward the pial surface and express several well-known markers of NSCs, including SOX2 and Musashi-1 (Msi1), and an astrocyte marker, glial fibrillary acidic protein (GFAP) (Barbosa et al., 2015; Dawley et al., 2012; Kaneko et al., 2000; Mchedlishvili et al., 2012; Naujoks-Manteuffel and Roth, 1989; Parish et al., 2007). In the intact urodele CNS, ependymoglia cells strongly express Msi1 and GFAP in the cell body and radial processes respectively (Dawley et al., 2012; Mchedlishvili et al., 2012).

During brain regeneration of urodele amphibians and teleosts, ependymoglia cells near the wound surface start to actively proliferate, migrate and differentiate, and these processes act to promote brain tissue regeneration (Amamoto et al., 2016; Kishimoto et al., 2012; Kroehne et al., 2011; Maden et al., 2013; März et al., 2011; Minelli et al., 1987; Okamoto et al., 2007). However, deciphering how ependymoglia cells give rise to the highly organized regenerated brain structure after excision of large parts of the intact brain remains a core unanswered question for understanding the variations of regeneration ability among vertebrates (Agata and Inoue, 2012).

During development, the identities of brain subregions become progressively defined according to embryonic neuroepithelial NSCs’ expression of sets of transcription factors (TFs), including homeobox genes or nuclear receptor genes, the expression level and timing of which are governed by morphogens secreted from secondary organizing centers in the brain, such as the isthmus (the mesencephalic-metencephalic boundary) and the floor and the roof plate (the right and left boundaries of the mesencephalic lobes) (Armentano et al., 2007; O'Leary et al., 2007; Vieira et al., 2010; Wurst and Bally-Cuif, 2001). Embryonic neuroepithelial NSCs transmit positional information (i.e. region-specific expression of TFs) radially to their progenies (Guillemot, 2007) and this system is accordingly a robust self-organizing system for maintaining the regional identity of each brain subregion, suggesting the possibility that brain regeneration in adult animals may also be subject to restraints of regional identity established in the developmental stage.

Like embryonic NSCs, adult ependymoglia cells and neurons in animals with regenerative ability show region-specific expression of several TFs, and their expression patterns are very similar to those in vertebrate embryos; for example, Pax7 expression in the dorsal mesencephalon (the optic tectum, OT) (Joven et al., 2013a,b; Schnapp et al., 2005). However, it remains largely unknown whether original brain structures can be regenerated if the brain loses an entire subregion containing a distinctive population of ependymoglia cells and thereby completely loses the specific positional information there (defined here as ‘large-scale brain injury’). It is speculated that in order to reconstruct the entire excised brain subregion, adult NSCs might show novel solutions for regaining the regional identity of the brain subregion rather than simply acting to increase the tissue volume by adding neuronal cells from corresponding domains of the pre-existing ependymal layer, by analogy to the properties of NSCs that have been reported in regeneration after a longitudinal incision in the zebrafish spinal cord (Kuscha et al., 2012). Thus, the establishment of a large-scale brain regeneration model in regenerative animals holds promise of providing insights into how adult NSCs rebuild highly regionalized brain structures from scratch.

Here, we used a unilateral OT excision method (modified from Minelli et al., 1987; Okamoto et al., 2007) in adult newts (*Pleurodeles waltl*) to characterize spatio-temporal dynamics of adult NSCs during large-scale brain regeneration. Using long-term observations over a period of 1 year, we unexpectedly found rostrocaudal differences in the regenerated cytoarchitecture of the OT, and this finding provides clues about how adult NSCs produce both perfect and imperfect brain structures in regeneration. This study further reveals certain similarities, and also certain differences, between development and regeneration; in contrast to development, which proceeds in successive steps from the neuroepithelium (Götz and Huttner, 2005; Smith and Schoenwolf, 1997), brain regeneration occurs under the constraint of ways of closing the wound by the local population of NSCs around boundary-domains of the mesencephalon.

## RESULTS

### Perfect and imperfect brain structural regeneration adjoined along the rostrocaudal axis

**Fig. 1.**
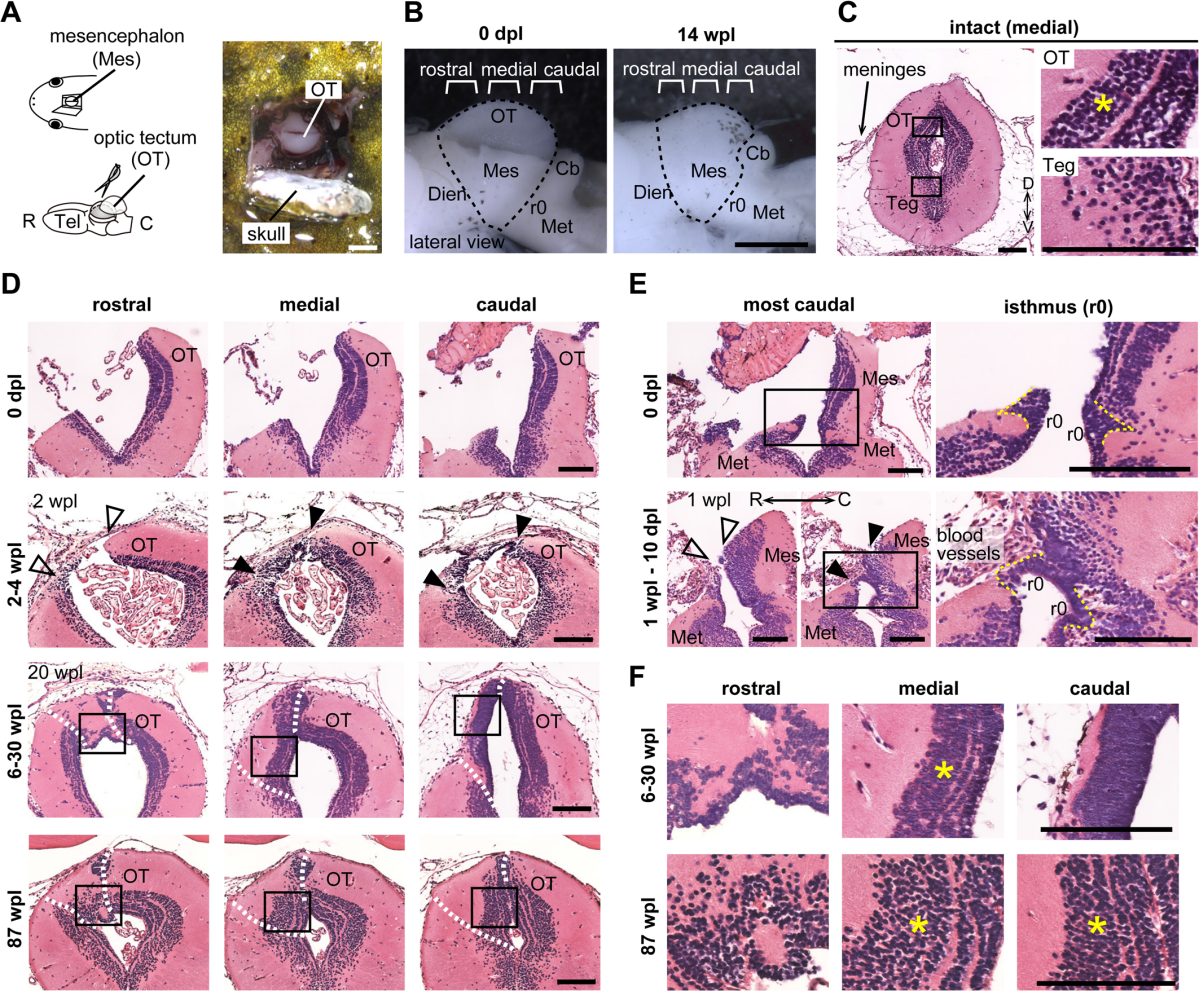
**Rostrocaudal differences in wound closure and laminar structure regeneration after unilateral OT excision.** (A) Left: schematic representation of the unilateral OT excision after opening a window in the skull. Right: dorsal view of the open window for surgery in living animals. (B) Lateral views of the lesioned newt brain at 0 dpl and at 14 wpl. Three different rostrocaudal levels represented in C–F are depicted. (C) Left: coronal section stained with HE showing the characteristic laminar structure in the OT of the intact mesencephalon, unlike in the Teg (*n*=4). Right: high magnification images of boxed regions in the left panel. (D) Timeline of unilateral OT regeneration at different rostrocaudal levels. Images were obtained from serial coronal sections at 0 dpl (*n*=4), 2–4 wpl (*n*=9), 6–30 wpl (*n*=8) and 87 wpl (*n*=3). Dotted lines indicate the dissected surfaces. Wound bridging by brain cells at 2–4 wpl was observed in the medial-to-caudal region (filled arrowheads), but not in the rostral region (open arrowheads). (E) The most caudal region of regenerating mesencephalon at 0 dpl (*n*=4) and at 1 wpl–10 dpl (*n*=4), showing that the earliest wound closing occurs around the isthmus (yellow dotted lines). Bottom left panels show two rostrocaudal levels of the most caudal mesencephalon. Wound bridging by brain cells at 1 wpl–10 dpl was observed around the isthmus (filled arrowheads), but not in more rostral adjacent sections (open arrowheads). Right panels are high magnification images of boxed regions in left panels. (F) High magnification images of boxed regions in D, showing that the rostral region has regenerated an abnormal arrangement of the cellular layer, whereas the medial-to-caudal region has completed progressive maturation of the laminar structure along the medial to caudal axis. Yellow asterisks indicate the tectal laminar structure. (A–F) Mes, Mesencephalon; OT, Optic tectum; Teg, Tegmentum; Tel, Telencephalon; Dien, Diencephalon; Cb, Cerebellum; Met, Metencephalon; r0, Rhombomere 0 (Isthmus); R, Rostral; C, Caudal; D, Dorsal; V, Ventral. Scale bars: 1 mm in A,B; 250 µm in C–F.

The OT is the bilateral dorsal subregion of the mesencephalon that acts as a visual center in non-mammalian vertebrate brains (Roth et al., 1990). To clarify whether regeneration occurs after excision of a whole unilateral OT, we performed surgery to excise approximately a quarter of the mesencephalon of a newt, *P. waltl* (Fig. 1A,B). *P.* *waltl* is a urodele amphibian that can easily be raised and handled in the laboratory, and that shows remarkable capacities to regenerate various organs (Hayashi et al., 2013, 2014). All experimental newts survived the surgery (*n*=71/71), and none showed any apparent abnormal behavior in walking, swimming or feeding. Within 3 months after surgery, the tubular structure was regenerated by injured brains (Fig. 1B, right).

To characterize regeneration-competent phenotypes, we then asked whether and when regenerating brains recovered tectal lamination, a hallmark of the OT (Fig. 1C), by performing serial cross-sectioning of samples fixed at different times of regeneration between 0 and 87 weeks post-lesion (wpl). Hematoxylin and Eosin (HE) staining showed recognizable patterns of cytoarchitecture of regenerating brains, which could be divided into five distinct stages separated by different time periods: 0 days post-lesion (dpl), 1 wpl–10 dpl, 2–4 wpl, 6–30 wpl, and 87 wpl (Fig. 1D-F).

Global wound closure occurred at 2–4 wpl (Fig. 1D), prior to the formation of the laminar structure at 6–30 wpl (Fig. 1D,F). During wound closure, the medial-to-caudal stumps were covered with cells and meninges, whereas in the rostral level, meninges alone seemed to tighten the open wound without regenerative cell invasion (Fig. 1D, arrowheads). Moreover, we found that in the earliest stage of wound closure at 1 wpl–10 dpl, the right and left sides of the unlesioned rhombomere 0 (r0; isthmus) were connected when wound surfaces of more rostral levels remained unconnected (Fig. 1E, arrowheads). At 6–30 wpl, the medial region recovered its lamination, while in the rostral region, lamination was unexpectedly disrupted, although many cell bodies and the white matter were regenerated (Fig. 1F). Immunohistochemistry for TuJ1 in the regenerating brain clearly demonstrated disrupted and orderly arrangement of axonal fibers in the rostral and the medial regions respectively (Fig. S1). In addition, we found that at 6–30 wpl, the caudal region seemed to show a delay in maturation, as indicated by the fact that we could not find clear lamination or a growing axonal layer there (Fig. 1F).

Over a period of 87 wpl (approximately 1.6 years), medial-to-caudal regions entirely recovered their lamination, while the rostral region remained unchanged from 6–30 wpl (Fig. 1D,F). These results indicated that by 87 wpl, the caudal-half region had completed its regeneration, which had gradually proceeded along the medial to caudal axis, whereas in contrast, the rostral region showed lower regenerative ability, with failure of lamination even at 1.6 years post-lesioning.

### The two rostrocaudal levels regenerated distinctive cellular subtypes but only the caudal level reconstructed the original cytoarchitecture

**Fig. 2.**
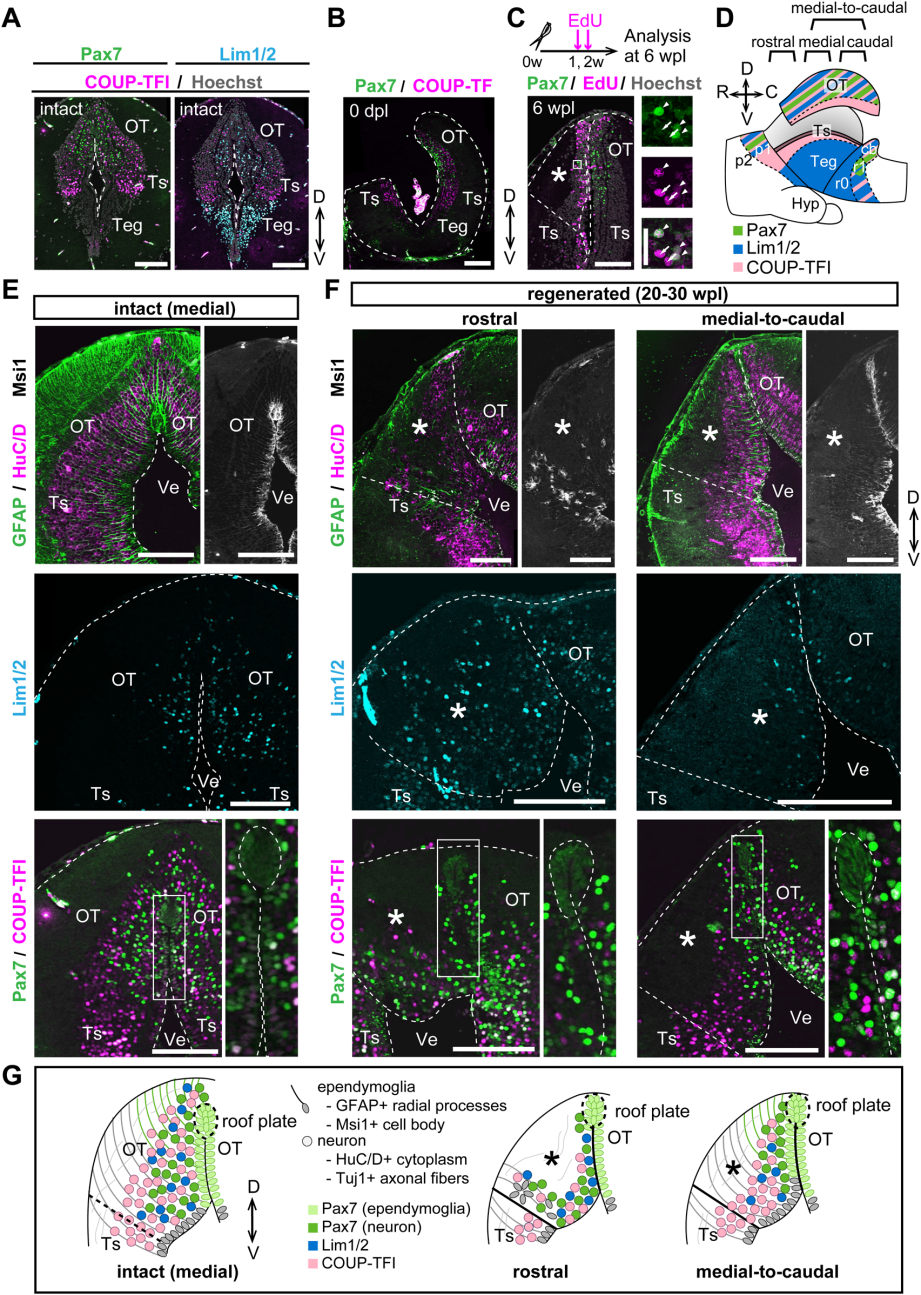
**Molecular characterization of the newt mesencephalic subregions and description of the regenerated cytoarchitecture in two rostrocaudal levels.** (A) Immunohistochemistry for Pax7, Lim1/2 and COUP-TFI on medial coronal sections of the intact newt mesencephalon defined as three major subregions (OT, Ts, Teg). The OT is characterized by heterogeneous expression of Pax7, COUP-TFI and Lim1/2 in the neuronal layer. (B,C) The Pax7-expressing unilateral OT was removed (B, *n*=4) and regenerated (C, *n*=3) after surgery. EdU-labeled newly generated cells were detected broadly in the injured area in 6 wpl regenerates, and some of them expressed Pax7 (positive, arrowheads; negative, arrows). (D) Schematic drawing of left-side view of the expression patterns of Pax7, Lim1/2 and COUP-TFI in the excised brain. (E,F) Immunohistochemistry for ependymoglia markers (GFAP, Msi1), a neuronal marker (HuC/D) and regional markers (Pax7, Lim1/2, COUP-TFI) on coronal sections of intact (E, *n*=3) and regenerated (F, *n*=3) mesencephalons reveals that the original cellular diversity in the OT was regenerated within 20–30 wpl at all rostrocaudal levels, whereas only the medial-to-caudal level recovered the apical-basal orientation of GFAP^+^ radial processes of Msi1^+^ ependymoglia cells like that in the intact mesencephalon (medial sections). (G) Schematic representation of immunohistochemical results. Asterisks indicate the regenerated area. Dotted lines indicate shapes of the mesencephalon, the roof plate and dissected surfaces. Ve, Ventricle; Ts, Torus semicircularis; p1-2, Prosomeres 1-2; Hyp, Hypothalamus; r1, Rhombomere 1. Scale bars: 250 µm in A,B,C (left panel) and E,F; 50 µm in C (right panels).

We then asked whether adult newts can regenerate the original neuronal and ependymoglial subtypes in the proper apicobasal/dorsoventral positions. To molecularly characterize the mesencephalic subregions, we first identified a set of TFs that were expressed in the mesencephalon in a region-specific manner along the dorsoventral axis in intact brains, through immunohistochemical screening using commercially available antibodies (data not shown). Using antibodies against three TFs: Pax7, Lim1/2 and COUP-TFI, we characterized the regional identity of the newt mesencephalon (Fig. 2A,E; Figs S2 and S3). Pax7 was discernibly expressed in the ependymal and neuronal layer of the adult newt OT, in accordance with a previous report (Joven et al., 2013a), and COUP-TFI was broadly expressed in the dorsal sensory area of the mesencephalon, namely the OT and the torus semicircularis (Ts). Lim1/2 was highly expressed in the neuronal layer of the motor tegmentum in the ventral mesencephalon, and expressed at a low level in that of the OT. The expression patterns of these three TFs showed good agreement with those in prior reports about various vertebrate embryos (Fedtsova and Turner, 2001; Matsunaga et al., 2001; Qiu et al., 1994), suggesting that the dorsoventral expression patterns of these TFs in the adult newt mesencephalon are similar to those in the embryonic vertebrate mesencephalon.

We then examined whether a unilateral Pax7^+^ OT could be successfully removed and regenerated after surgery. By using anti-Pax7 and anti-COUP-TFI antibodies to detect the respective antigens as dorsal markers, we demonstrated successful removal of the Pax7^+^ OT from the ipsilateral mesencephalon, leaving the COUP-TFI^+^ Ts (Fig. 2B), as illustrated in Fig. 2D. Lineage tracing experiments with the nucleotide analog 5-ethyl-2′-deoxyuridine (EdU) revealed that the excised region was filled with numerous EdU^+^ newly generated cells, and that subpopulations of these cells expressed Pax7 (Fig. 2C), indicating that several neuronal subtypes, including Pax7^+^ neurons, were regenerated by 6 wpl.

Next, we investigated whether the original cellular subtypes were regenerated in specific apicobasal/dorsoventral positions of the brain. In the intact brain, apical lining ependymoglia cells expressed Msi1 in their cell bodies and extended their GFAP^+^ radial processes toward the brain surface, whereas HuC/D^+^ pan-neurons were closely packed in the parenchymal region (Fig. 2E). In the OT, Pax7 is expressed both in ependymoglia cells and a subpopulation of neurons, however, expression levels of Pax7 protein and cellular properties differed by cellular subtypes: neurons in the Msi1^−^ parenchymal region were recognized as Pax7^high^ scattered cells, while ependymoglia cells in the Msi1^+^ ependymal layer were recognized as Pax7^low^ closely packed cells (Fig. S3). Thus, intact OTs comprised three neuronal subtypes (Pax7^high^, COUP-TFI^+^ and Lim1/2^+^ neurons) and Pax7^low^ ependymoglia cells that included cells composing the dorsal roof plate (Fig. 2E,G).

At 20–30 wpl, the medial-to-caudal region showed recovery of the correct radial topography of GFAP^+^ radial processes of Msi1^+^ ependymoglia cells and the HuC/D^+^ neuronal layer that comprised Pax7^high^, COUP-TFI^+^ and Lim1/2^+^ neurons (Fig. 2F). In contrast, the rostral region showed a disorganized structure displaying abnormal arrangement of Msi1^+^ ependymoglia cells, the GFAP^+^ radial processes and HuC/D^+^ neuronal layer, although Lim1/2^+^, Pax7^high^ and COUP-TFI^+^ neurons were regenerated and the cluster of Pax7^low^ ependymoglia cells in the dorsal roof plate was exceptionally well formed along the entire rostrocaudal axis (Fig. 2F). We summarized the regenerated cytoarchitecture of both rostral and medial-to-caudal levels in Fig. 2G.

### Pax7^low^ ependymoglia cells were immediately regenerated, prior to Pax7^high^ neurons, along the entire rostrocaudal axis

**Fig. 3.**
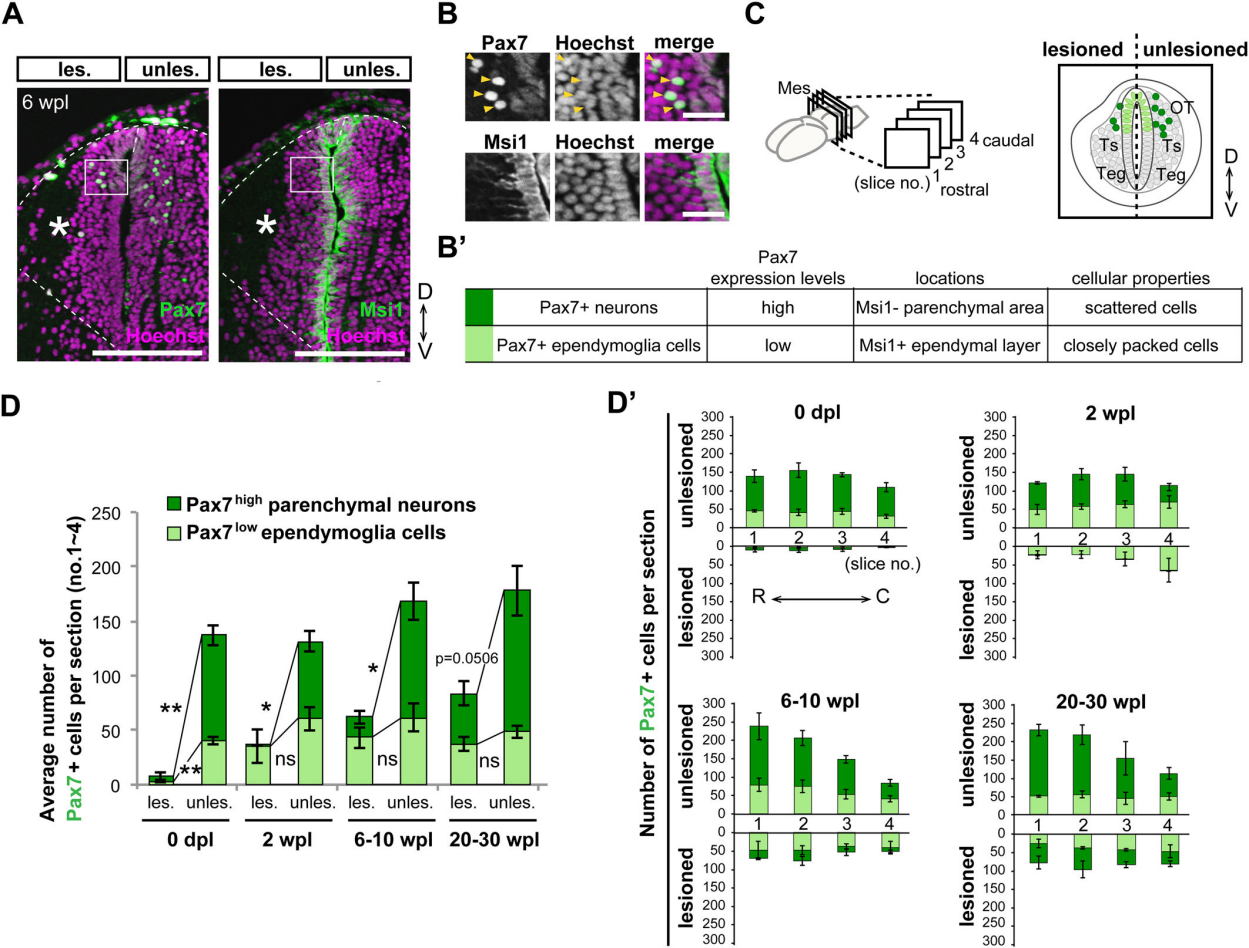
**All rostrocaudal levels rapidly regenerate Pax7^low^ ependymoglia cells and gradually recover Pax7^high^ neurons.** (A–B′) Immunohistochemistry for Pax7 and Msi1 on adjacent coronal sections of the regenerating brain at 6 wpl, showing that Pax7^+^ ependymoglia cells and neurons in the OT have different expression levels of Pax7 and distinct locations and morphologies. Dotted lines indicate shapes of the mesencephalon and dissected surfaces. Asterisks indicate the regenerated area. Boxed regions as depicted in A. (B) Pax7^high^ neurons exhibiting a rounded morphology were found in the Msi1^−^ parenchyma (arrowheads), while Pax7^low^ ependymoglia cells displaying epithelial morphologies were found in the Msi^+^ ependymal layer. These evaluation criteria are summarized in B′. (C) For counting cells, coronal sections were obtained from four different rostrocaudal levels (nos. 1-4, from rostral to caudal). By using adjacent coronal sections for Msi1 immunostaining, we counted the number of Pax7^high^ neurons and Pax7^low^ ependymoglia cells according to the evaluation criteria. Right panel depicts a typical image of sectioning. (D) Stacked bar chart shows the average number of Pax7^low^ ependymoglia cells and Pax7^high^ neurons in four coronal sections of regenerating OT [lesioned (les.) and unlesioned (unles.) side] at 0 dpl, 2 wpl, 6–10 wpl and 20–30 wpl (*n*=3 animals, mean±s.e.m., ns>0.05, *P≤0.05, **P≤0.01; Welch's *t*-test). (D′) Stacked bar chart shows rostrocaudal and right and left distributions of the number of Pax7^low^ ependymoglia cells and Pax7^high^ neurons summarized in D (*n*=3 animals, mean±s.e.m.). Scale bar: 250 µm in A; 50 µm in B.

To investigate the regeneration process of OT-specific ependymoglia cells and neurons in detail, we evaluated the timing of recovery of Pax7^+^ ependymoglia cells and Pax7^+^ neurons in the lesioned and unlesioned mesencephalic lobes in the same individual. When we counted the number of Pax7^+^ ependymoglia cells and Pax7^+^ neurons, we judged these cell types not only by expression levels of Pax7, but also by adjacent sections stained with the antibody against Msi1 (Fig. 3A,B) and additionally by their cellular properties (Fig. 3B′). Quantification of the average number of Pax7^high^ neurons and Pax7^low^ ependymoglia cells in coronal sections (Fig. 3C) revealed rapid regeneration of Pax7^low^ ependymoglia cells within 2 wpl, (*n*=3, *P*=0.248; Fig. 3D). In contrast, the Pax7^high^ neuronal population was virtually absent from the regenerating brain at 2 wpl (*n*=3, *P=0.0167; Fig. 3D), and was gradually increased over 20–30 weeks (*n*=3, *P*=0.0506; Fig. 3D). Rapid regeneration of Pax7^low^ ependymoglia cells and subsequent differentiation into the Pax7^high^ neurons occurred in all of the rostrocaudal planes (Fig. 3D′), indicating that the production of OT-specific Pax7^low^ NSCs and their differentiation are not strongly dependent on the position along the rostrocaudal body axis.

### Active proliferation at early stages in the caudal level caused Msi1^+^ ependymoglia cells to be in a wound-covering formation

**Fig. 4.**
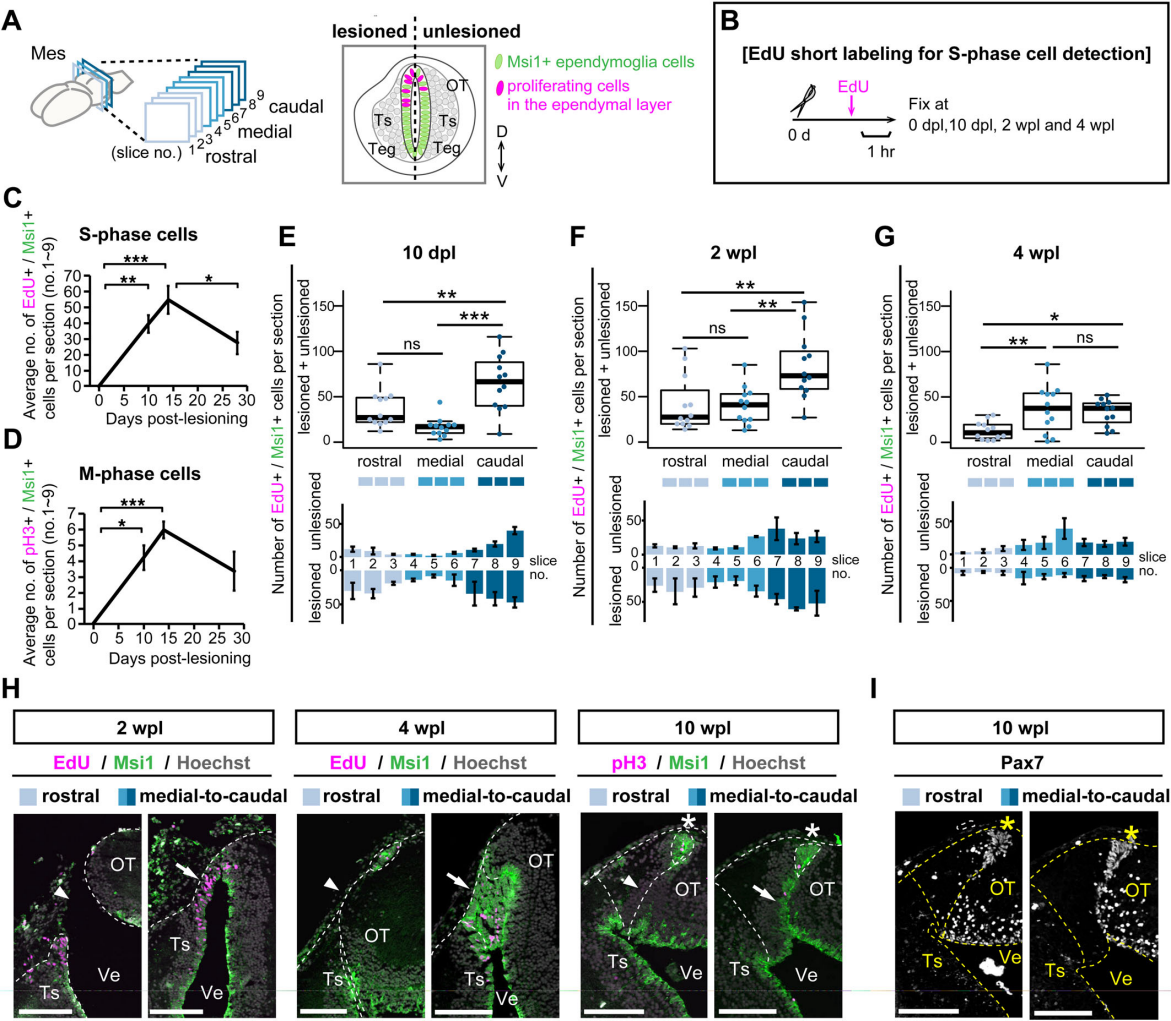
**Spatio-temporal patterns of cell proliferation reflect caudal-biased wound closure by ependymoglia cells.** (A) For counting cells, coronal sections were obtained from nine different rostrocaudal levels (nos.1–3; rostral, nos.4–6; medial, nos.7–9; caudal). Right panel shows a typical image of sectioning. (B) Schema of *in vivo* EdU labeling for S-phase cell detection. (C) Quantification of the average number of EdU^+^/Msi1^+^ S-phase or pH3^+^/Msi1^+^ M-phase proliferating ependymoglia cells in entire coronal sections (nos.1–9) of 0 dpl, 10 dpl, 2 wpl and 4 wpl regenerates (*n*=4 animals, mean±s.e.m.) revealed that proliferation actively occurred around 2 wpl. (E-G) Scatter and box plots of EdU^+^/Msi1^+^ S-phase cells at three different rostrocaudal levels in 10 dpl, 2 wpl and 4 wpl regenerates (upper panels, *n*=4 animals, *n*=3 sections) revealed that robust proliferation of S-phase cells occurred in the caudal mesencephalon at 10 dpl (E) and 2 wpl (F). S-phase cells were counted separately in the lesioned and unlesioned side of a mesencephalic section depicted as in A (lower panels, *n*=4 animals, mean±s.e.m.). (H) Immunohistochemistry for Msi1 on coronal sections at rostral and medial-to-caudal levels of the regenerating brain at 2, 4, and 10 wpl. Proliferating cells were detected by EdU incorporation or immunostaining with pH3 antibody. Arrowheads indicate abnormal gap between newly formed and the pre-existing ependymal layer, while in contrast, arrows indicate the contiguous ependymal layer bridging wound surfaces. (I) Immunohistochemistry for Pax7 on coronal sections adjacent to H (10 wpl). Asterisks indicate Pax7 expression in the Msi1^+^ roof plate. Dotted lines indicate shapes of the mesencephalon, dissected surfaces and the roof plate structure. ns>0.05, *P≤0.05, **P≤0.01, ***P≤0.001; one-way ANOVA with post-hoc Tukey's multiple comparison test. Scale bars: 250 µm.

Why did the rostral and caudal levels show different abilities of brain architectural regeneration, despite the fact that the number of OT-specific ependymoglia cells recovered to the original number in the entire mesencephalon? To answer this, we investigated the spatio-temporal proliferation pattern of Msi1^+^ ependymoglia cells during regeneration by performing analysis of cell proliferation in sequential coronal sections, by monitoring of EdU incorporation to mark S-phase cell, or monitoring of mitosis marker pH3 (Fig. 4A,B).

Proliferation was detected as the number of EdU^+^/Msi1^+^ S-phase cells or pH3^+^/Msi1^+^ M-phase cells in each coronal section. When we calculated the average numbers of these cells, we found that both S-phase- and M-phase-Msi1^+^ ependymoglia cells were not detected at 0 dpl, and that their numbers increased between 10 dpl and 2 wpl, and then EdU^+^ S-phase-cells were decreased by 4 wpl (Fig. 4C,D). Strikingly, by comparing three rostrocaudal levels around the time of the proliferative phase at 10 dpl and 2 wpl, we found that the number of S-phase ependymoglia cells in the caudal level was significantly higher than the numbers in the medial and the rostral levels (Fig. 4E,F). Quantification of M-phase ependymoglia cells revealed a similar trend of high numbers in the caudal region at 10 dpl, in comparison to the medial and the rostral levels (Fig. S4A). At the end of the proliferative phase, i.e. at 4 wpl, proliferating cells were evenly distributed throughout the medial-to-caudal levels (Fig. 4G; Fig. S4C).

Immunohistochemical analysis of regenerating brains revealed that the spatial arrangement of proliferative ependymoglia cells was also dramatically different between the rostral (Fig. 4H, arrowheads) and medial-to-caudal (Fig. 4H, arrows) levels. At 2 wpl, proliferating Msi1^+^ ependymoglia cells bridged the lesioned and unlesioned mesencephalic lobes only in the medial-to-caudal levels. After the entire rostrocaudal wound surfaces were closed to recover a tubular structure of the brain at 4 wpl, medial-to-caudal regenerated Msi1^+^ ependymoglia cells changed their histological properties from a uniformly packed stratified or folded cell layer into a typical single ependymal layer during 4–10 wpl (Fig. 4H, arrows). In contrast, in the rostral level at 4–10 wpl, regenerated Msi1^+^ ependymoglia cells were only localized in the roof plate region, leading to the formation of an abnormal gap between the newly formed and the previously existing Msi1^+^ ependymal layer (Fig. 4H, arrowheads), i.e. to incomplete structural regeneration. However, in 10-week regenerates as well as 20–30-week regenerates (Fig. 2F), the Msi1^+^/Pax7^+^ roof plate was regenerated in both rostrocaudal levels (Fig. 4H,I, asterisks).

The above results indicate that during unilateral OT regeneration, ependymoglia cells in the caudal level underwent a burst of proliferation compared with those in the rostral-to-medial levels, which seems to contribute to the formation of a continuous Msi1^+^ ependymal layer, including the roof plate. Whereas in the rostral level, the tubular topology of the brain was recovered without extensive proliferation of ependymoglia cells, thus resulting in abnormal regeneration that had a gap between the newly reconstructed roof plate and the previously existing ependymal layer.

### The earliest differentiated cells were found in the caudoventral mesencephalon, around the isthmus

**Fig. 5.**
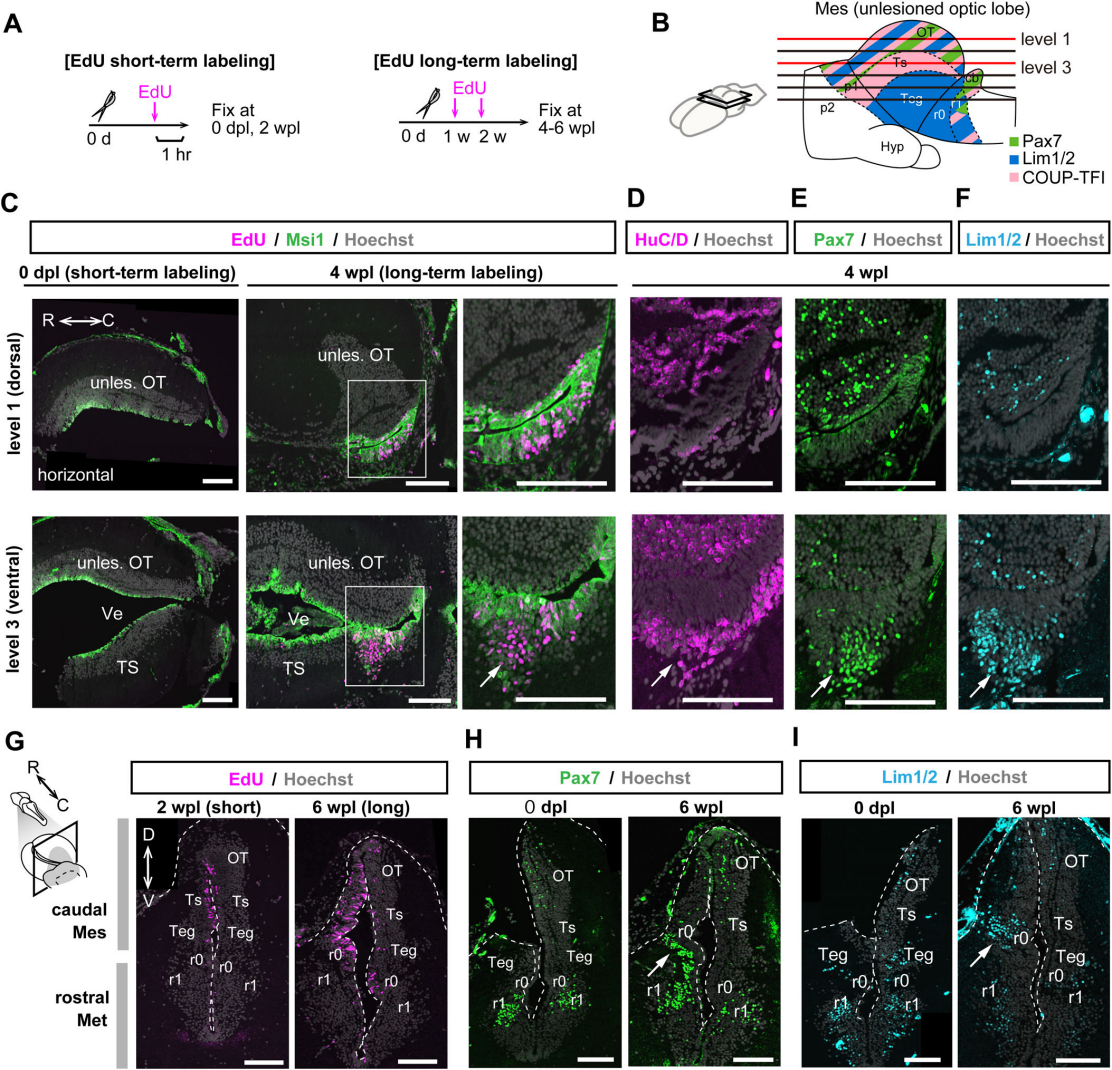
**Early differentiation occurs around the isthmus.** (A) Experimental designs of EdU short-term labeling for detection of proliferating cells, and EdU long-term labeling for cell fate tracing of proliferated cells. (B) Schematic representation of section levels used for immunohistochemistry and EdU detection (level 1 and level 3 for panels C–F). The results from six horizontal levels are shown in Fig. S5. (C) EdU detection and immunohistochemistry for Msi1 on horizontal sections at two dorsoventral levels of the lesioned brain at 0 dpl and 4 wpl, showing that Msi1^low^/EdU^+^ cells can be found predominantly in the caudoventral mesencephalon at the early regeneration stage (arrow). In contrast, caudodorsal mesencephalon maintained undifferentiated ependymoglia cells (Msi1^high^/EdU^+^) at the same stage. (D-F) Caudodorsal and caudoventral sections adjacent to C (4 wpl) showed that Msi1^low^/EdU^+^ cells in the caudoventral mesencephalon (C-F, arrows) expressed a pan-neural marker HuC/D (D), Pax7 (E) and/or Lim1/2 (F). In contrast, Msi1^high^/EdU^+^ undifferentiated ependymoglia cells in the caudodorsal mesencephalon expressed Pax7 (E), and did not express HuC/D (D) and Lim1/2 (F). (G) Coronal sections of the caudal mesencephalon at 2 wpl and 6 wpl, showing that EdU-labeled cells were actively proliferating around the r0 (isthmus) between 2 wpl and 6 wpl. (H,I) Coronal sections of the caudal mesencephalon at 0 dpl and 6 wpl, showing that Pax7^+^ (H) and Lim1/2^+^ (I) early-differentiated cells were observed in the caudoventral region, around the isthmus. Arrows indicate the same caudoventral position around the isthmus, where we found early-differentiated cells. Dotted lines indicate shapes of the mesencephalon. Scale bars: 250 µm.

Cellular differentiation would also be crucial for regeneration of complex brain structures. To visualize actively differentiating regions after the proliferation phase, which occurs between 10 dpl and 4 wpl (Fig. 4), we examined the distribution of newly generated EdU^+^ cells in 4–6 wpl regenerates after pulse labeling with EdU at 1 and 2 wpl (Fig. 5A). Serial horizontal sections revealed that EdU^+^ cells emerged as the predominant population in the caudal mesencephalon (Fig. 5B,C; Fig. S5). In the caudodorsal edge, EdU^+^/Msi1^high^ ependymoglia cells maintained an undifferentiated state until at least 4 wpl (Fig. 5C) and expressed Pax7 (Fig. 5E). In contrast, in the caudoventral region, we found the EdU^+^/Msi1^low^ parenchymal region (Fig. 5C, arrow) occupied by HuC/D^+^ early-differentiated cells expressing Pax7 and Lim1.2 (Fig. 5D-F; Fig. S5C, arrows).

Coronal images corresponding to the region in which we found early-differentiated cells revealed that Pax7 and Lim1/2 were expressed in subpopulations of parenchymal cells near rhombomere 0 (r0; isthmus) (Fig. 5G-I, arrows). It is known that r0 lacks Pax7 expression, whereas rhombomere 1 (r1) in the metencephalon strongly expresses Pax7 in newts (Joven et al., 2013a). We therefore concluded that early-differentiated Pax7^+^ cells are r1 cells in the metencephalon resulting from a hyperplastic response (Fig. S5B,B′, arrows), but we could not determine the region to which the early-differentiated Lim1/2^+^ cells belonged.

### Caudoventral ependymoglia cells produced multiple kinds of progenies along the rostrodorsal direction

**Fig. 6.**
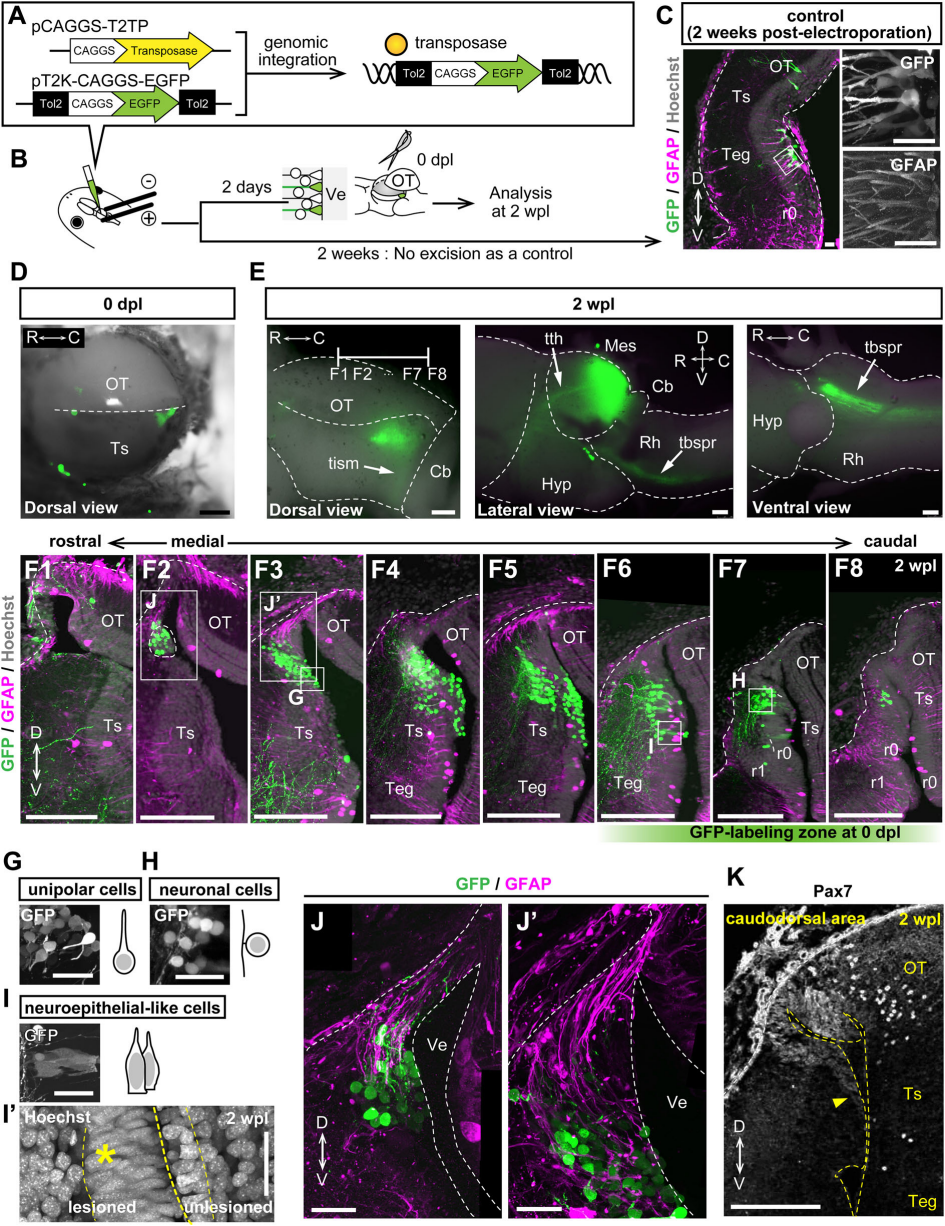
**Caudoventral ependymoglia cells serve as a driving force for wound closure and simultaneously produce mature efferent neurons at a very early stage.** (A,B) Schema of electroporation to label caudoventral ependymoglia cells in the ipsilateral mesencephalon, and of the experimental protocol after electroporation. By using an EGFP reporter plasmid and a Tol2 transposase expression vector (A), it was observed that EGFP signals could be strongly sustained in progenies of initially labeled ependymoglia cells, presumably because the reporter gene was integrated into the genome after electroporation. (C) Immunohistochemistry for GFP and GFAP on coronal sections of an unlesioned control at 2 weeks after electroporation. High magnification images of boxed regions show GFP-labeled cells have a radial morphology typical of GFAP^+^ ependymoglia cells (right panels). (D,E) Images of a lesioned mesencephalon after electroporation at 0 dpl (D) and 2 wpl (E) observed using a fluorescence stereoscopic microscope. The caudoventral stump in the ipsilateral mesencephalon was labeled by GFP at 0 dpl, and was raised up to the level of the contralateral optic tectum at 2 wpl. Arrows indicate the tractus tecto-isthmicus (tism), tractus tectothalamicus (tth) and tractus tectobulbospinalis rectus (tbspr) projections that were regenerated within 2 weeks. (F1–F8) Immunohistochemistry for GFP and GFAP on serial coronal sections of the electroporated regenerating brain at 2 wpl. Corresponding positions are depicted in E, and F6-F8 are approximate positions labeled with GFP at 0 dpl. (G-I) High magnification images and schematic images of boxed regions in F3, F7 and F6 respectively, reveal that GFP^+^ cells can be divided into three morphologically different cell types: unipolar cells (G), neuronal cells (H) and neuroepithelial-like cells (I). (I′) Hoechst staining of the regenerating brain at 2 wpl revealed that the ependymal layer of the lesioned side of the mesencephalon had a neuroepithelial-like morphology (asterisk). (J,J′) High magnification images of boxed regions in F2 and F3, showing that GFAP^+^ radial processes of ependymoglia cells covered the wound surface and GFP^+^ unipolar cells migrated in the caudoventral-to-rostrodorsal direction along processes. (K) Pax7 expression was localized in the dorsal domain of the regenerated ependymal layer. Arrowhead indicates the expression boundary of Pax7. Rh, rhombencephalon. Scale bars: 250 µm in D–F8, K; 50 µm in C,G–J′.

We then asked whether caudoventral ependymoglia cells have the potential to regenerate bona fide OT cells, not just hypertrophic scars. To address this question, we electroporated a CAG promoter-driven GFP construct under the tol2 system into the caudoventral stump to visualize the cell fate of ependymoglia cells in the caudoventral region (Fig. 6A,B). The *in vivo* electroporation method has been well established to be useful for specific labeling of ventricular ependymoglia cells of adult newts (*Notopthalmus viridescens*) with high specificity (Berg et al., 2011). We have also performed preliminary experiments of *in vivo* electroporation using *P. waltl* and showed that the majority (>95%) of first labeled cells were ventricular ependymoglia cells possessing long radial processes, assessed by morphological criteria (Fig. S6).

In unlesioned controls, GFP signals were hardly detectable by stereomicroscopic examination at 2 weeks after electroporation (data not shown), because GFP-labeled ependymoglia cells remained in the apical area maintaining the undifferentiated state (Fig. 6C). In contrast, in lesioned brains (Fig. 6D, *n*=3), GFP signals were broadly detected in the caudal mesencephalon at 2 wpl (Fig. 6E). In addition, we detected axonal GFP expression (Fig. 6E, arrows) that accorded with major efferent tectal projections as previously reported in the intact newt mesencephalon, namely the tractus tecto-isthmicus (tism), tractus tectothalamicus (tth) and tractus tectobulbospinalis rectus (tbspr) (Nieuwenhuys et al., 1998). These observations suggest that within 2 wpl, progenies of GFP-labeled caudoventral ependymoglia cells or themselves are able to produce long-distance efferent projections which are possibly involved in OT-specific neuronal circuits.

To further characterize descendants of GFP-labeled ependymoglia cells in the surrounding tissue environment, we performed immunohistochemical analysis on serial coronal sections using antibodies against GFP and GFAP (Fig. 6F1-F8). In regenerates, GFP-labeled cells were morphologically different from typical ependymoglia cells (Fig. 6C), and could be classified into the following three morphological types: unipolar cells, neuronal cells and neuroepithelial-like cells (Fig. 6G-I, respectively). Unipolar cells and neuronal cells were widely distributed in medial-to-caudal levels (Fig. 6F2-F8), not being limited to the caudal pole of the ependymal layer that was first labeled (Fig. 6F6-F8), suggesting that these cells migrated after lesioning. Unipolar cells were clustered together in a relatively inner area of the gray matter, including the ependymal layer (Fig. 6F3,G). In addition, we found that GFAP^+^ radial processes bridged the gap between wound surfaces and formed a scaffold for GFP^+^ unipolar progenies migrating in the caudoventral-to-rostrodorsal direction (Fig. 6J,J′). GFP-labeled neurons were found in the outermost layer of the gray matter and extended long axons into the pre-existing axonal layer in a parallel fashion (Fig. 6F4-F7,H). This structure indicates that these neurons are efferent neurons, and their axonal projections are displayed in Fig. 6E.

The last morphological type of GFP-labeled cells, neuroepithelial-like cells, were emerged in the ependymal layer of the caudal mesencephalon during regeneration (Fig. 6I′; Fig. S7). The regenerated ependymal layer was already regionalized as distinguished by the dorsal expression of Pax7 at 2 wpl (Fig. 6K). We found that on the lesioned side, neuroepithelial-like cells formed a thick ependymal layer in comparison with the unlesioned control side (Fig. 6I′). However, we noted that in the caudal pole, the thick ependymal layer was present on both sides that seemed to form the segmental curved shape bridging the right-and-left mesencephalic lobes together at several dorsoventral levels (Fig. S7).

## DISCUSSION

Ependymoglia cells serve as a cell source for CNS regeneration upon brain injuries in regenerative animals (Becker and Becker, 2015). However, the sequential process of brain regeneration whereby ependymoglia cells produce the highly organized cytoarchitecture of the adult brain has remained elusive. Here, we investigated large-scale brain regeneration in adult newts, and long-term observation over the period of 1 year revealed spatio-temporal ependymoglia behavior that led to both perfect and imperfect structural regeneration within an intraindividual animal.

### NSC arrangement and kinetics enabling proper structural brain regeneration

Three models have previously been used for studies of relatively large-scale brain regeneration in highly regenerative adult animals, namely, partial excision of the dorsal telencephalon in axolotl (Amamoto et al., 2016), partial excision of the cerebellum in zebrafish (Kaslin et al., 2017), and partial removal of tissue within the unilateral OT of newt (Minelli et al., 1987; Okamoto et al., 2007). The results obtained using the first two of these models have revealed unexpected limitations in structural brain regeneration, i.e. adult axolotl and zebrafish regenerate either insufficient original cellular subtypes or aberrant axial cellular arrangements (Amamoto et al., 2016; Kaslin et al., 2017). The newt studies, in contrast, have shown that upon partial removal of the unilateral OT, neural tissues including cholinergic neurons, the retinotectal projection area, and the laminar structure in the OT, are regenerated within 6 months (Minelli et al., 1987; Okamoto et al., 2007), implying that adult newts may have a strong capacity for regeneration of a structurally normal brain. To investigate the details of such structural brain regeneration from adult NSCs, a description of the ultimately achieved cytoarchitecture of the regenerated brains is necessary. However, it remains unknown whether perfect structural regeneration that combines recoveries of brain regionalization and the original tissue structure occurs in adult newts. Furthermore, why regenerative animals show different regeneration-competent phenotypes depending on the animal species remains a largely unanswered question.

In this study, we excised a whole unilateral OT of adult newts, and confirmed the excised region by molecularly defining the newt mesencephalic subregions using region-specific cellular markers (Pax7, COUP-TFI and Lim1/2) and ependymoglial makers (GFAP and Msi1) (Fig. 2; Figs S2 and S3). Using these markers in combination with HE staining, we found that upon unilateral OT excision, newts can regenerate the laminar structure and their original cellular subtypes, the Pax7^+^/COUP-TFI^+^/Lim1/2^+^ mixed neuronal population and Pax7^+^ ependymoglia cells, in parts of the regenerated region within 20–30 weeks (Figs 1 and 2). Moreover, electroporation results clearly showed regeneration of long-distance efferent projections (Fig. 6E). This is the first report, to our knowledge, of complete structural brain regeneration restoring the original cellular subtypes in the proper apicobasal/dorsoventral positions, in addition to the long-distance neuronal circuit, after a large-scale brain excision in an adult vertebrate.

Most importantly, structural regeneration occurred only in the caudal half of the mesencephalon, while in contrast, the rostral level regenerated a disorganized brain structure that lacked the laminar structure and contiguous ependymal layer despite regenerating the same neuronal subtypes as the caudal level (Figs 1 and 2). Similarly, a limitation of structural brain regeneration that results in a lack of either regeneration of original cellular subtypes or their proper axial cellular arrangement has been reported in axolotl telencephalons and zebrafish cerebellums after the underwent the partial removal of the respective brain subregion (Amamoto et al., 2016; Kaslin et al., 2017). In the present study, different regeneration-competent phenotypes along the rostrocaudal axis within the same individual allowed us to clarify the process necessary for structural brain regeneration, especially by focusing on the NSC behavior.

Wound closure in brain regeneration has been defined as an initial process of regeneration that fills in injuries by proliferation and/or migration using surrounding NSCs, and failure to regenerate has been explained by the absence of wound-closing ability (Endo et al., 2007; Yoshino and Tochinai, 2004), but how resident ependymoglia cells reestablish or fail to reestablish the brain patterning through initial wound closure has not yet been described. We show here that perfect or imperfect regeneration of brain patterning can be recognized at early stages, by comparing the wound closure process between two rostrocaudal levels (Figs 1 and 4). We found that although both axial levels completed wound closing and regenerated the Pax7^+^ roof plate structure, only the caudal level regenerated Pax7^+^ NSCs (ependymoglia cells) that were juxtaposed with the pre-existing ependymal layer (Fig. 4). Our cell counting results showed that during 10 dpl–2 wpl, cell proliferation activities of Msi1^+^ ependymoglia cells in the caudal mesencephalon were clearly higher than those in the rostral-to-medial region (Fig. 4; Fig. S4), suggesting that the caudal NSC activity is likely to be a driving force for regenerating the contiguous ependymal layer found in the medial-to-caudal mesencephalon.

Indeed, by using *in vivo* electroporation, we elucidated parts of the wound closure process by which progenies of the caudoventral ependymoglia cells migrated in the caudoventral-to-rostrodorsal direction through cell migration and/or cell proliferation (Fig. 6). At the cellular level, we found that morphologically three types of cells were involved in early stages of brain regeneration: unipolar cells, neuronal cells and neuroepithelial-like cells (Fig. 6G-I). An interesting finding regarding wound closure is that unipolar progenies migrate to bridge the wound surfaces along bundles of GFAP^+^ radial processes of ependymoglia cells (Fig. 6F1-F8,J,J′). The mechanism by which radial processes are arranged, which is presumably required for determining the direction of migration of cells, and the fate of unipolar cells themselves remain to be further addressed. Overall, our findings indicate that possible causes of brain regeneration failure derive not only from the absence of wound closure (Endo et al., 2007) but also from an improper arrangement of ependymoglia cells during recovery of a tubular topology of the brain. In contrast, perfect structural regeneration occurs under the condition that regenerated ependymoglia cells bridge the wound to recover a continuous ependymal layer at later stages (Fig. 4). However, there are still many unanswered questions about how several cell types, including ‘unipolar cells’, play cooperative roles in the initial wound closure and regeneration.

### Similarities and differences between brain regeneration and development

Neuroepithelial NSCs are one of the most important cell types in vertebrate brain patterning during development, since they convey the positional identity to their progenies, and in particular, neuroepithelial secondary organizing centers at specific boundaries between brain subdomains organize the specification of each brain subregion identity via secretion of morphogens (Briscoe and Small, 2015; Vieira et al., 2010; Wurst and Bally-Cuif, 2001). In the embryonic mesencephalon, there are three secondary organizer areas involved in axial cell fate determination: the isthmus, the dorsal roof plate and the ventral floor plate.

Similarly to mesencephalic development, we found that neuroepithelial-like tissue appeared over a wide range of the regenerating brain at 2 wpl, especially on the lesioned side and the caudal pole of the mesencephalon (Fig. 6I′; Fig. S7). Previous studies have demonstrated that a neuroepithelial type of ependymoglia cells plays an important role in CNS regeneration by expanding the regenerating field of the axolotl spinal cord (Rodrigo Albors et al., 2015) or by predominantly generating the missing neuronal subtypes in the zebrafish cerebellum (Kaslin et al., 2017). We also observed a dorsoventral segmentation in the neuroepithelial-like layer in the regenerating mesencephalon, by sectioning it into 80 µm thick coronal sections (Fig. S7). However, in this study we could not find detectable molecular markers to visualize the dorsoventral polarity on the mesencephalic ependymal layer (except Pax7). Intriguingly, different adhesion properties of distinctive dorsoventral levels in the embryonic mesencephalon account for the formation of autonomous units of these precursors (Li et al., 2005). Further work will be needed to identify fine-grained regionalization by using markers for cell adhesion molecules as the candidates for the responsible factors.

The most interesting findings of the present work are two prominent behaviors of regenerating cells around the roof plate and the isthmus. First, the lesioned Pax7^+^ roof plate exhibited a self-organizing regenerative ability to regain its original structure along the dorsal midline of the mesencephalon, regardless of whether other regenerated areas eventually recovered the perfect OT structure or not (Figs 2 and 4). It was not only in the roof plate; rapid recovery of Pax7^+^ ependymoglia cells by 2–10 wpl occurred in all the rostrocaudal levels and then Pax7^+^ neurons were gradually regenerated in the dorsal area (Fig. 3). Second, in contrast to gradual differentiation of Pax7^+^ OT neurons, we observed the earliest differentiation of Pax7^+^ r1 and Lim1/2^+^ mesencephalic cells around the regenerating ipsilateral isthmus (Fig. 5), suggesting that the isthmic region may provide a neurogenic niche at a very early stage of unilateral OT regeneration.

In this study, how regenerated neuronal subtypes were finally mixed together in the OT remained unanswered. To answer this question, the dynamics of neuronal regeneration is worth considering. Curiously, the neuronal regeneration sequence bears some resemblance to development. In OT development in chicks, efferent neurons are the first differentiated cell types produced from neuroepithelial cells (Gray and Sanes, 1991, 1992; Reiner and Karten, 1982). This is similar to our electroporation results in that both observations have revealed the early emergence of efferent neurons and a neuroepithelial type of cells during OT development or regeneration (Fig. 6). Moreover, tectal lamination, which is formed by the invasion of unmyelinated fibers (Roth et al., 1990), appears as a rostral-to-caudal wave of maturation in the development of various non-mammalian vertebrates (LaVail and Maxwell Cowan, 1971; Schmidt and Roth, 1993; Straznicky and Gaze, 1972; Thomas et al., 2006), in accord with our results obtained from long-term staging of OT regeneration, which proceeded in a rostral-to-caudal direction over the course of more than 1 year, and the caudal pole of the mesencephalon, including the isthmus and the roof plate, formed the laminar structure at the end (Fig. 1).

**Fig. 7.**
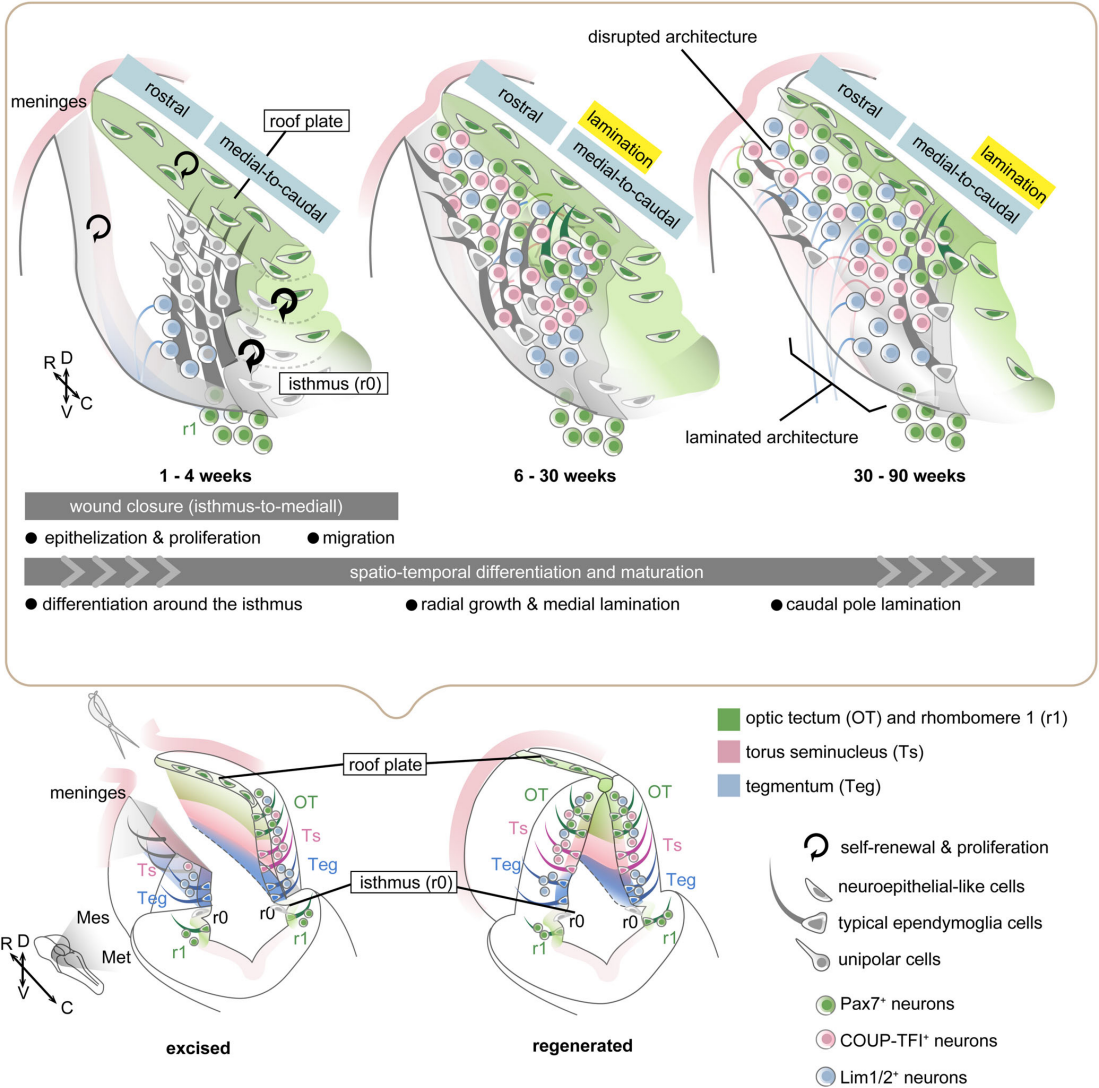
**Model of newt brain regeneration.** Schematic drawings of regenerating brains viewed from the same angle illustrate different regeneration stages upon unilateral OT excision, showing that both perfect and imperfect structural regeneration along the rostrocaudal axis can be attributed to wound closing, differentiation and maturation processes. Epithelialization and proliferation (1–4 weeks): Wound surfaces are immediately covered with meninges (Fig. 1). The wound closure with regenerative cell invasion initially occurs around the isthmus, and then occurs along the caudal to medial axis (Fig. 1). Regenerative proliferation starts in Msi1^+^ ependymoglia cells, mostly at the caudal level (Fig. 4). The original size of the region-specific ependymoglia cell population (Pax7^+^) is roughly recovered (Fig. 3), and in particular, the Pax7^+^ roof plate structure is self-organized within 4 wpl (Fig. 4). Bilateral mesencephalic domains are fused by neuroepithelial-like ependymoglia cells at each dorsoventral level of the caudal pole of the mesencephalon (Fig. S7). Migration and differentiation around the isthmus (1–4 weeks): Unipolar cells migrate along radial processes to cover the caudal-to-medial level but not the rostral level (Fig. 6). At the same time, efferent tectal neurons (presumably Lim1/2^+^ cells) and Pax7^+^ r1 neurons in the metencephalon are rapidly differentiated around the isthmus (Figs 5 and 6). Radial growth and medial lamination (6–30 weeks): Regenerated ependymal layer in the caudal-to-medial level undergoes radial growth (Figs 1 and 4). The rostral level lacks the apical Msi1^+^ ependymal layer that is fused to the pre-existing one, and thus regenerates disrupted brain architecture except for the self-organized roof plate (Figs 2 and 4). Caudal pole lamination (30–90 weeks): Different neuronal subtypes (Pax7^+^, Lim1/2^+^ and COUP-TFI^+^) are juxtaposed to form the original OT composition (Fig. 2). Maturation of tectal laminar structure occurs via rostral (medial) to caudal progression, whereas the rostral level retains disrupted architecture (Figs 1 and 2).

Such striking similarities between development and regeneration strongly suggest that in the caudal half of the mesencephalon, structural brain regeneration shares cell-intrinsic mechanisms that govern vertebrate CNS development, after proliferation and proper arrangement of NSCs. By contrast, the following point differs: in development, neuroepithelial NSCs uniformly distribute throughout the primordial brain and proceed to successive steps of brain morphogenesis (Götz and Huttner, 2005; Smith and Schoenwolf, 1997). Whereas, in OT regeneration, ‘modules’ of boundary-domain NSCs around the roof plate and the isthmus play an active part in the local self-organization, proper wound closure and early differentiation, thus only their neighboring fields will be able to regain the original cytoarchitecture of the brain (Fig. 7). Altogether, we provide a detailed description of large-scale brain regeneration in adult newts, as summarized in Fig. 7, showing that ways of regeneration-specific wound closure can determine the outcome of structural regeneration of the brain cytoarchitecture. These findings show that that regenerative events start locally in the boundary-domain of the brain, and this could yield important insights for unravelling the cellular basis of various regenerative abilities of adult vertebrate brains.

## MATERIALS AND METHODS

### Animals

Sexually mature Iberian ribbed newts, *P. waltl*, were originally obtained from Dr T. Hayashi (Tottori University, Tottori, Japan). The experiments were carried out on adult newts that were about 12 cm long, had been raised from eggs and had metamorphosed after half a year to 1 year in our laboratory. The animals were kept in plastic containers in dechlorinated tap water at 24–26°C under a 12/12 h light/dark cycle, mimicking natural conditions, and were fed more than twice a week. All animal procedures were carried out using protocols approved by the Animal Care and Use Committee of Kyoto University (Kyoto, Japan).

### Excision of the unilateral optic tectum

Newts were anesthetized with 0.2% MS222 (Sigma-Aldrich) and placed on the stage of a stereomicroscope. A 4×4 mm region of the scalp and skull above the mesencephalon was cut on three sides with a disposable surgical knife, making an open ‘window’ with a hinge. After opening this window, the meninges were torn with fine forceps and blood was rinsed off with 1×Steinberg solution supplemented with 100 U/ml penicillin and 100 µg/ml streptomycin (Meiji Seika Pharma, Tokyo, Japan). The right and left optic lobes were detached using a tungsten needle. The left OT (a quarter of the mesencephalon) was excised with ophthalmic scissors and the cut surface was trimmed, and then the top of the scalp and skull were restored to their original position. The post-surgery animals were kept on wet paper towels for 1 week (until the scalp wound was closed), and then transferred to normal breeding water, where they started to feed normally.

### Immunohistochemistry

Anesthetized newts were perfusion-fixed with 4% paraformaldehyde in phosphate-buffered saline (PBS), and then the brains were removed and postfixed in the same fixative at 4°C overnight. To prepare cytosections, the fixed samples were immersed in 20% sucrose in PBS, embedded in OCT compound (Sakura Finetek Japan, Tokyo, Japan), and cut into 12 µm thick coronal or horizontal sections. For free-floating sections in Figs 3 and 6 (except for Fig. 6K) and Figs S5B, S6 and S7, the fixed samples were washed with PBS and then cut into 80 µm thick coronal sections.

The sections were rinsed in TPBS (PBS with 0.1% Triton X-100) for 15 min and blocked with 10% normal goat serum for 1 h. For some antibodies (anti-COUP-TFI and anti-Lim1/2) that were used with tyramide signal amplification (TSA), sections were pretreated with 0.3% H_2_O_2_ in PBS for 30 min to deactivate endogenous peroxidases. After the sections were rinsed twice with TPBS, they were incubated with TNB buffer (0.1 M Tris-HCl, pH 7.5; 0.15 M NaCl; and 0.5% blocking reagent) for 30 min for blocking. Sections were incubated with the primary antibodies described below, which were diluted in TPBS, at room temperature overnight: mouse anti-Pax7 [Developmental Studies Hybridoma Bank (DSHB); 1:600], rabbit anti-GFAP (Dako, Santa Clara, CA, USA; Z0334; 1:500), rat anti-Musashi-1 (eBioscience, Waltham, MA, USA; 14-9896; 1:600), mouse anti-HuC/D (Molecular Probes, Waltham, MA, USA; MP21271; 1:500), mouse anti-Tuj1 (Covance, Princeton, NJ, USA; MMS-435P; 1:500), rabbit anti-phospho-Histone H3 (pH3) (Millipore; 06-570; 1:1000), or rat anti-GFP [Medical & Biological laboratories (MBL), Nagoya, Japan; D153-3; 1:700]. After sections were rinsed three times with TPBS, they were incubated with the following secondary antibodies containing 10 ng/ml Hoechst 33342 (Molecular Probes, Eugene, OR, USA) in TPBS for 2 h: Alexa Fluor series (Invitrogen; Alexa Fluor 488, 594, or 633; 1:500) or HRP-conjugated goat anti-mouse IgG (H+L) (Life Technologies, Carlsbad, CA, USA; 1:1500). HRP-conjugated secondary antibody was detected using a TSA kit (PerkinElmer, Waltham, MA, USA). Sections were mounted on glass slides in Fluoro-KEEPER Antifade Reagent (Nakalai Tesque, Kyoto, Japan).

### Histology

The anesthetized newts were perfusion-fixed with 4% paraformaldehyde in PBS, and subsequently the head portion was immersion-fixed with 75% methanol/25% acetic acid fixative at 4°C overnight. Heads were decalcified in Osteosoft (Merck, Darmstadt, Germany) and embedded in paraffin. Serial paraffin sections of 10 µm thickness were made from the brains of intact newts and newts at different times after the surgery, and stained with HE. Hematoxylin (Muto Pure Chemicals, Tokyo, Japan) diluted 1:10 and Eosin Y (Merck) were used for the staining.

### EdU long-term tracing

One hundred microliters of EdU (1 mg/ml in PBS) (Tokyo Chemical Industry, Tokyo, Japan) was injected twice into the abdominal cavity (at 1 wpl and 2 wpl), and the anesthetized animals were euthanized at 4 wpl or 6 wpl. After cryosectioning into 12 µm slices, EdU incorporation was detected using a Click-iT EdU Imaging Kit (Invitrogen), and then immunohistochemistry for double staining was performed as described above.

### Detection of cell proliferation

For detecting S-phase cells at various time points, 1 mg/ml EdU (Tokyo Chemical Industry, 100 µl) was injected into the abdominal cavity at 0 or 10 dpl, or 2 or 4 wpl. Newts were euthanized 1 h after EdU injection for ‘short-term labeling’, and perfusion fixed and postfixed with 4% paraformaldehyde in PBS. Cryosections were prepared as described above for immunohistochemistry. To detect M-phase cells, anti-pH3 antibody was used.

### Assessment of cell fate by electroporation

A ‘window’ was opened in the skull of anesthetized newts as described above. Then plasmid DNA solution [a mixture of pT2K-CAGGS-EGFP and pCAGGS-T2TP (Kawakami and Noda, 2004; Urasaki et al., 2006) containing 1 mg/ml of each plasmid in PBS and 0.05% Fast Green] was injected into the third ventricle of the brain, and electroporated in the left-caudal ependymal layer twice with five square-wave pulses of 50 V/cm (10 ms pulse length, 999 ms pulse interval) applied using an electroporator CUY21 (Nepa Gene, Chiba, Japan). Two days after electroporation, the unilateral left OT was excised. Images of electroporated brains were obtained using a fluorescence stereomicroscope (Leica Microsystems, Wetzlar, Germany; M205 FA).

### Image acquisition, image processing, cell count and statistical analysis

Images of immunolabeled sections and HE staining sections were obtained using an FV10i confocal microscope (Olympus, Tokyo, Japan) and an Axiovert 200M microscope (Carl Zeiss, Oberkochen, Germany), respectively. In immunolabeled sections, 12 µm thick sections were photographed as single z-plane images, while 80 µm thick sections were photographed as Z-stack-constructed images using FV10-ASW software (Olympus). Original images were stitched together by using Image Composite Editor (Microsoft) or ImageJ (NIH) and processed by linear adjustments with ImageJ and Photoshop (Adobe). For improving viewability of multi-color images, triple and double immunolabeled sections including Hoechst detection were digitally processed via the following procedures using Photoshop (Adobe): (1) A single fluorescent channel for Hoechst was copied on another layer and converted into gray in RGB channels. (2) To reduce the excessive brightness when merged with other immunofluorescent signals, the fill percentage of the Hoechst layer was changed from 100% to 30%. (3) Another one or two immunofluorescent signals, excluding the Hoechst signal, were processed on the layer for which blending mode was to ‘screen’. (4) A single image was obtained by displaying two layers for Hoechst and other immunofluorescent signals.

All experimental animals were randomly obtained from the F1 generation of the same parents, to avoid bias derived from the age of the animals at the time of surgeries. Cells were manually counted using ImageJ. Statistical analysis was performed using an unpaired two-tailed Student's *t*-test with Welch correction for two groups of observations, and one-way ANOVA followed by Tukey's post-hoc test for multiple comparisons. All immunohistochemistry and histology results were obtained from experiments repeated at least three times using different animals.

## Supplementary Material

Supplementary information

First Person interview
