# Supplementary material for: Spatio-temporal neural stem cell behavior leads to both perfect and imperfect structural brain regeneration in adult newts
Source: Biol Open. 2018 Jun 14;7(6):bio033142. doi: 10.1242/bio.033142 (PMC6031346; doi:10.1242/bio.033142)
Supplement: Supplementary information [file biolopen-7-033142-s1.pdf]

Supplementary information

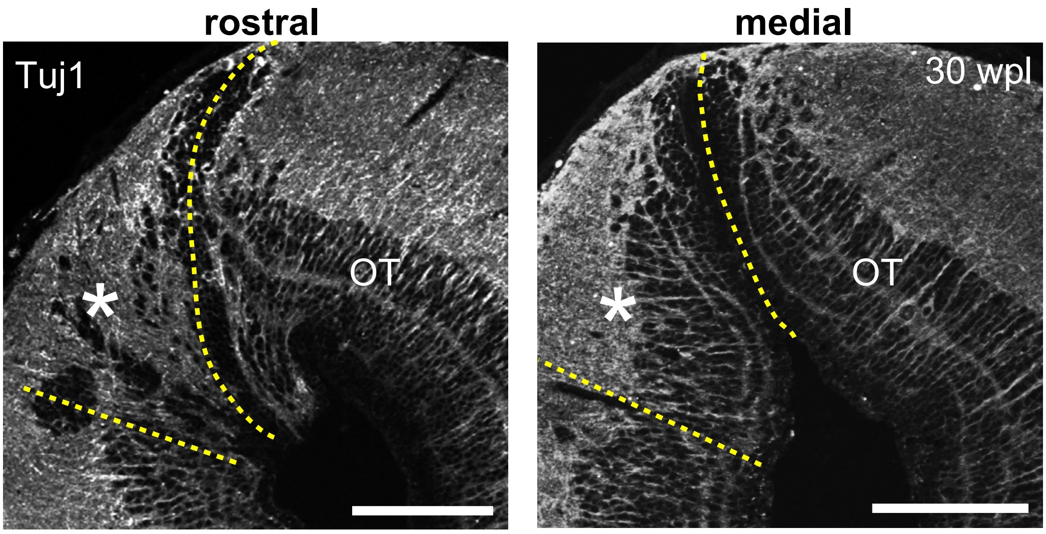

Fig. S1 (related to Fig. 1). Arrangement of axonal fibers in the rostral and medial regenerating mesencephalon.

Immunohistochemistry for a neuronal marker, Tuj1, on coronal sections of the regenerating mesencephalon at different rostrocaudal levels, showing different regeneration-competent phenotypes of the axonal structure in the regenerated tissue (asterisks). By 20-30 wpl (n=3), the medial level regenerated the laminar structure (right), whereas the rostral level regenerated disrupted axonal structure (left). Yellow dotted lines indicate dissected surfaces. Scale bars: 250 μm.

Biology Open • Supplementary information

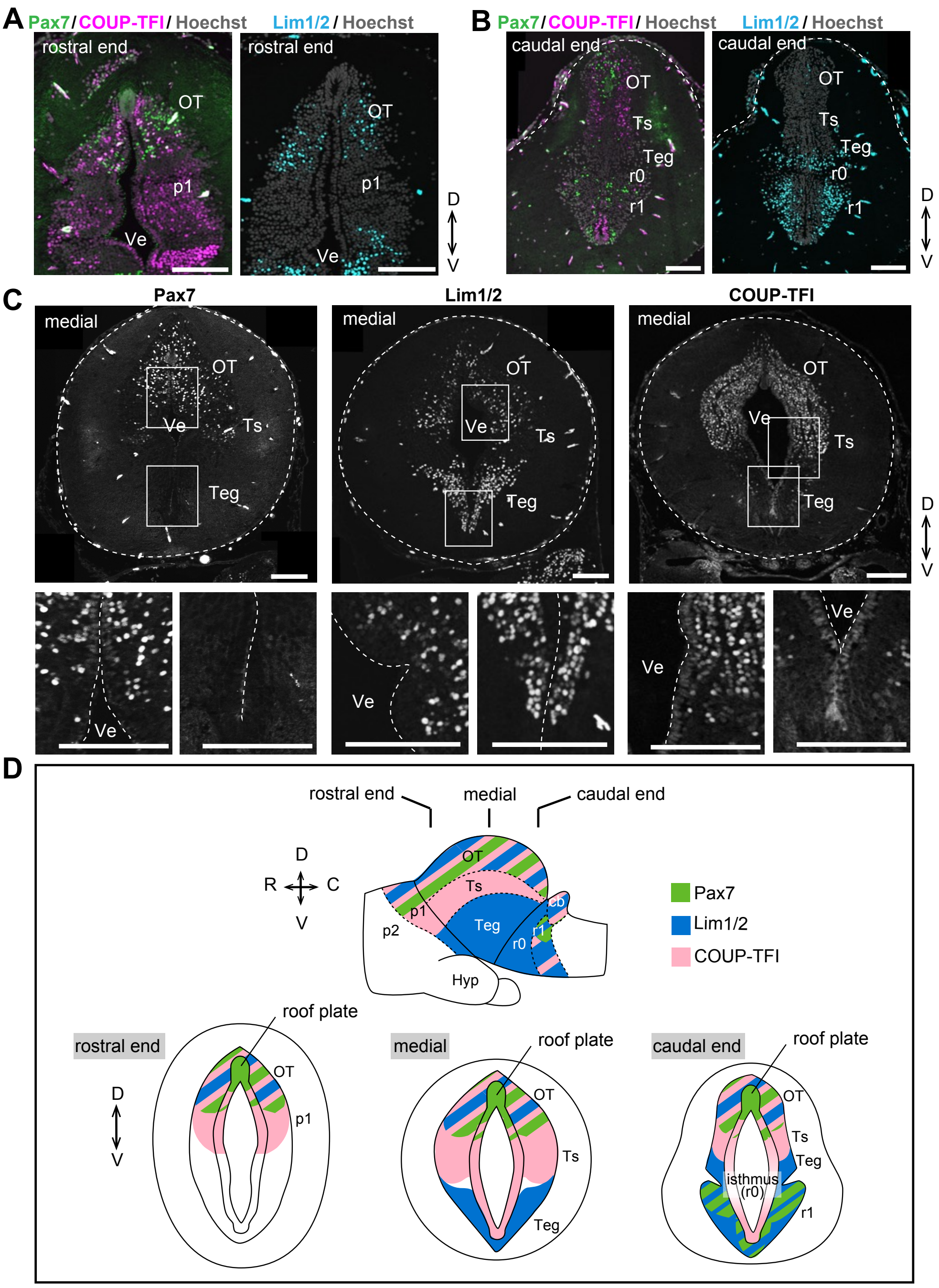

Fig. S2 (related to Fig. 2). Molecular characterization of the newt mesencephalic subregions.

(A) Immunohistochemistry for Pax7, COUP-TFI and Lim1/2 on coronal sections of the rostral (A) and caudal (B) ends of the mesencephalon. (C) Expression of Pax7, Lim1/2 and COUP-TFI in the medial mesencephalon shows that Pax7 is selectively expressed in the OT, which includes the ependymal layer. (D) Schematic representation of expression patterns of the three transcription factors in the intact mesencephalon of adult newts. Lateral view (upper) and corresponding coronal sections at different rostrocaudal levels (lower). Scale bars: 250 µm.

Biology Open • Supplementary information

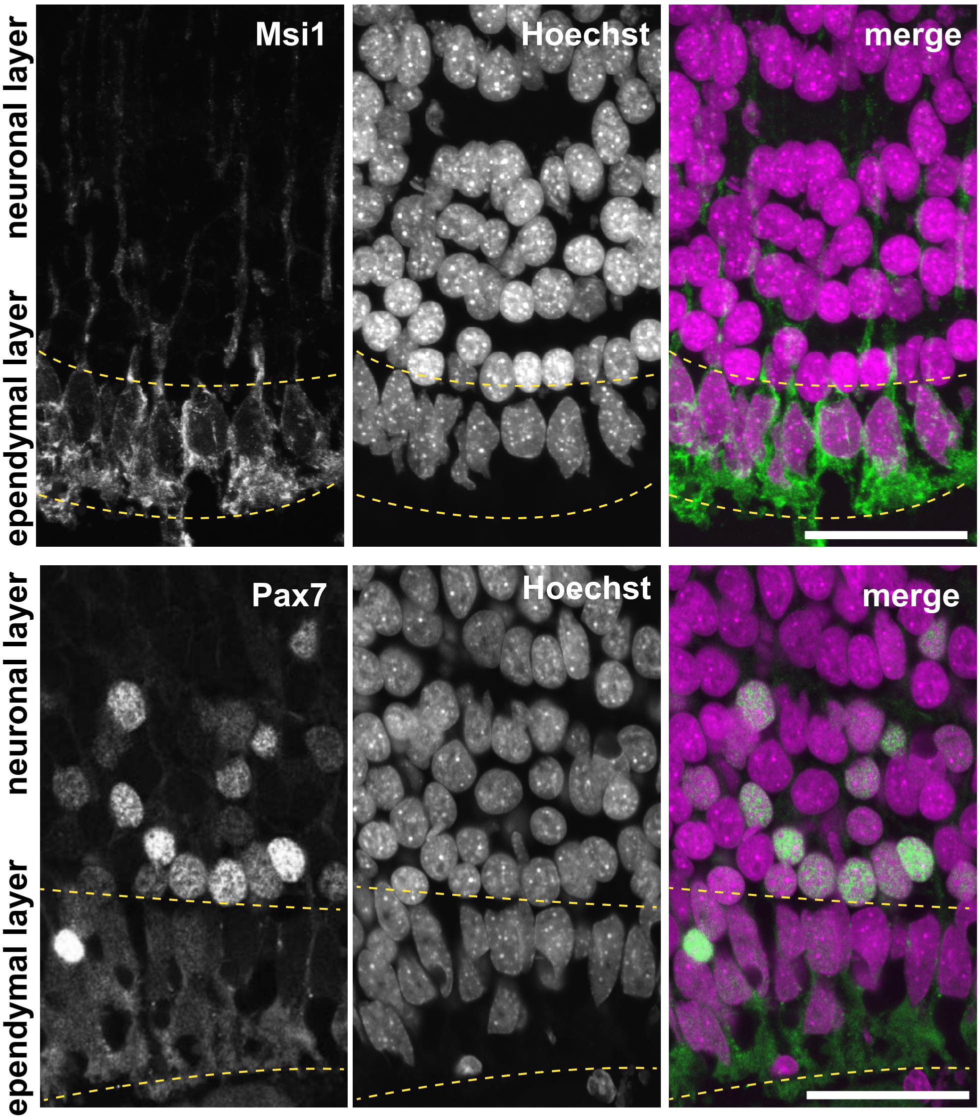

Fig. S3 (related to Fig. 2). Molecular and morphological characterization of Pax7^{+} cells.

Immunohistochemistry for an ependymoglia marker, Msi1, and Pax7 on adjacent coronal sections of the intact mesencephalon, showing that Msi1^{+}/Pax7^{+} ependymoglia cells and Msi1^{-}/Pax7^{+} neurons in the OT have different expression levels of Pax7, distinct locations and morphologies. Pax7^{high} neurons in the Msi1^{-} parenchymal region exhibited a rounded morphology, while Pax7^{low} ependymoglia cells in the Msi1^{+} ependymal layer displayed irregular epithelial cell morphology. Yellow dotted lines indicate the ependymal layer. Scale bars: 50 μm.

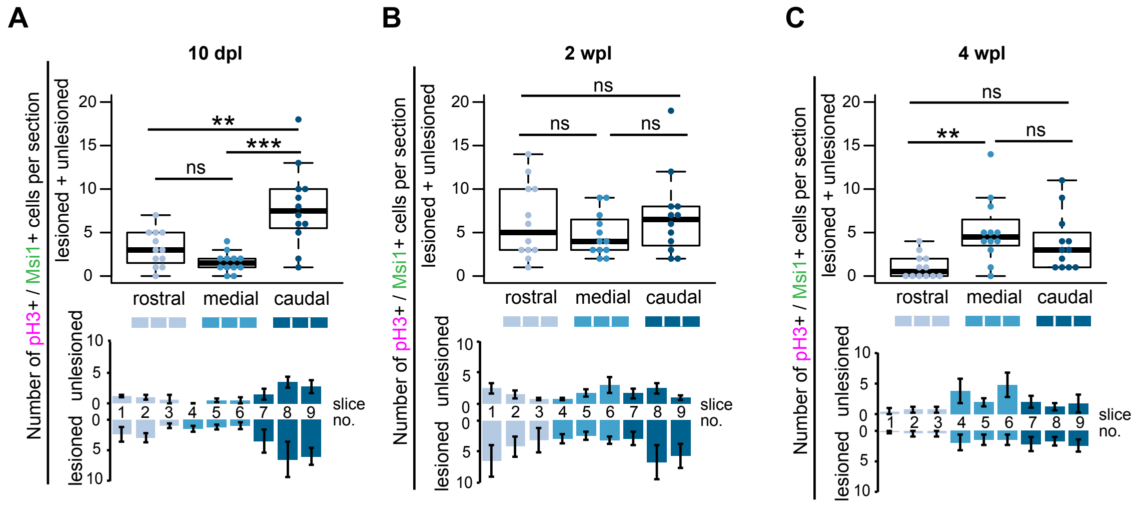

Fig. S4 (related to Fig. 4). Spatio-temporal distribution patterns of M-phase cells along the rostrocaudal axis.

(A-C) Scatter and box plots of $pH3^{+}/Msi1^{+}$ M-phase cells at three different rostrocaudal levels in 10-day, 2-week and 4-week regenerates (upper panels, n=4 animals, n=3 sections) revealed that robust proliferation of M-phase cells occurred in the caudal mesencephalon at 10 dpl. M-phase cells were separately counted in the lesioned and unlesioned side of a mesencephalic section depicted as in Fig. 4A (lower panels, n=4 animals, mean ± SEM). ns>0.05, *p≤0.05, **p≤0.01, ***p≤0.001; one-way ANOVA with post-hoc Tukey's multiple comparison test.

Biology Open • Supplementary information

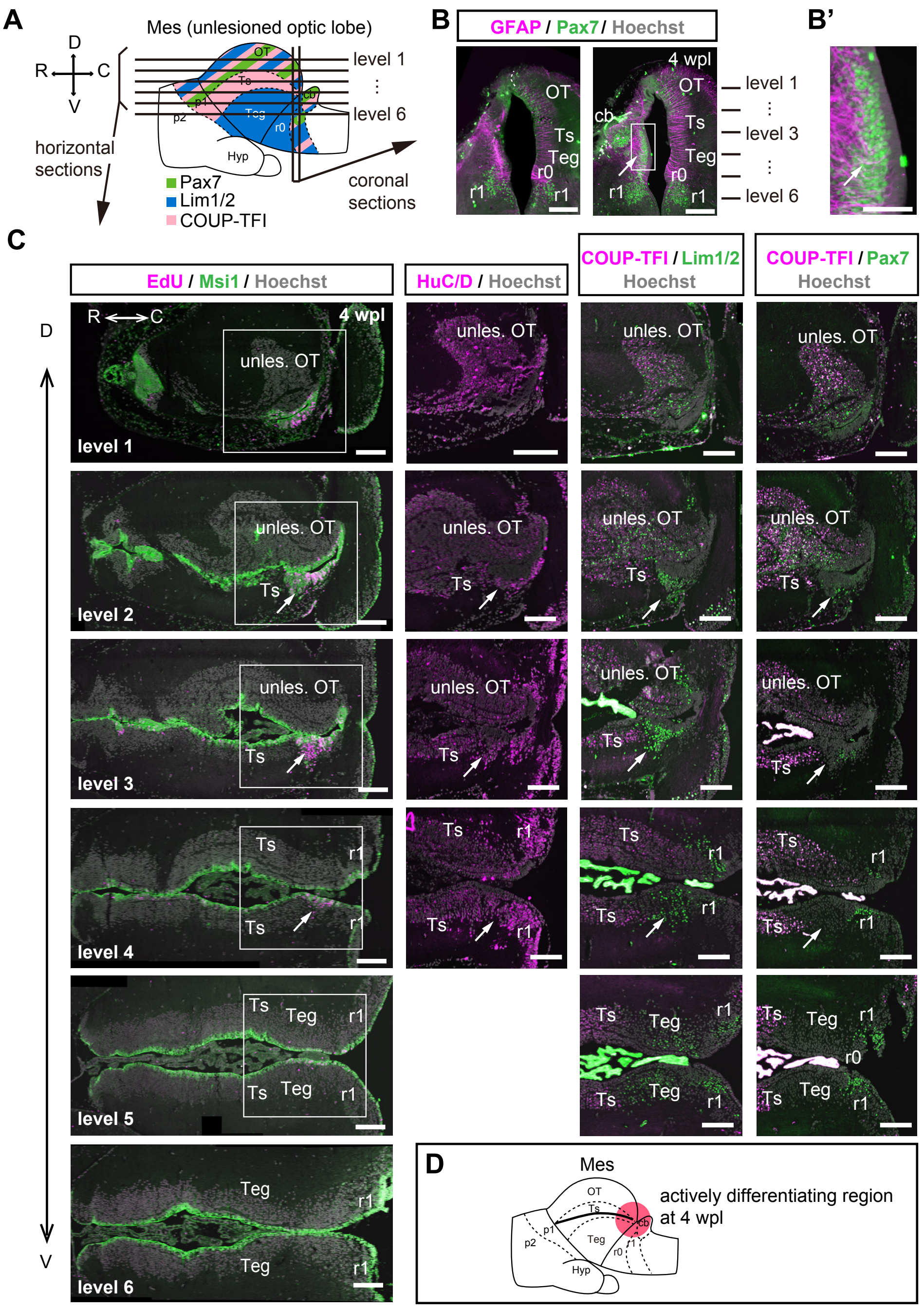

Fig. S5 (related to Fig. 5). Neuronal subtypes of the differentiated cells at the early stage of regeneration.

(A) Schematic representation of section levels used for immunohistochemistry and EdU detection (panels B,C). (B) Immunohistochemistry for GFAP and Pax7 on adjacent coronal sections of the regenerating brain at 4 wpl (left, rostral; right, caudal), showing that a mass of Pax7$^+$ cells was observed in the most caudoventral part of the mesencephalon, around the ipsilateral isthmus. Corresponding horizontal levels

Biology Open • Supplementary information

represented in C are shown in the right of the panels (B). High magnification image of the boxed region in B, showing that Pax7^{+} cells were localized in the parenchymal region (B'). (C) EdU detection and immunohistochemistry for Msi1, HuC/D, COUP-TFI, Lim1/2 and Pax7 on serial horizontal sections of the regenerating brain at 4 wpl revealed that the actively differentiating region (arrows) comprised Msi1^{low}, HuC/D+, Lim1/2^{+} and/or Pax7^{+} cells. Right three panels are serial horizontal sections adjacent to left panels. (D) Schematic representation of the actively differentiating region at 4 wpl. Arrows indicate the corresponding region, where we found early-differentiated cells. Unles. OT, unlesioned optic tectum. Scale bars: 250 μm in B, C; 100 μm in B'.

Biology Open • Supplementary information

Biology Open (2018): doi:10.1242/bio.033142: Supplementary information

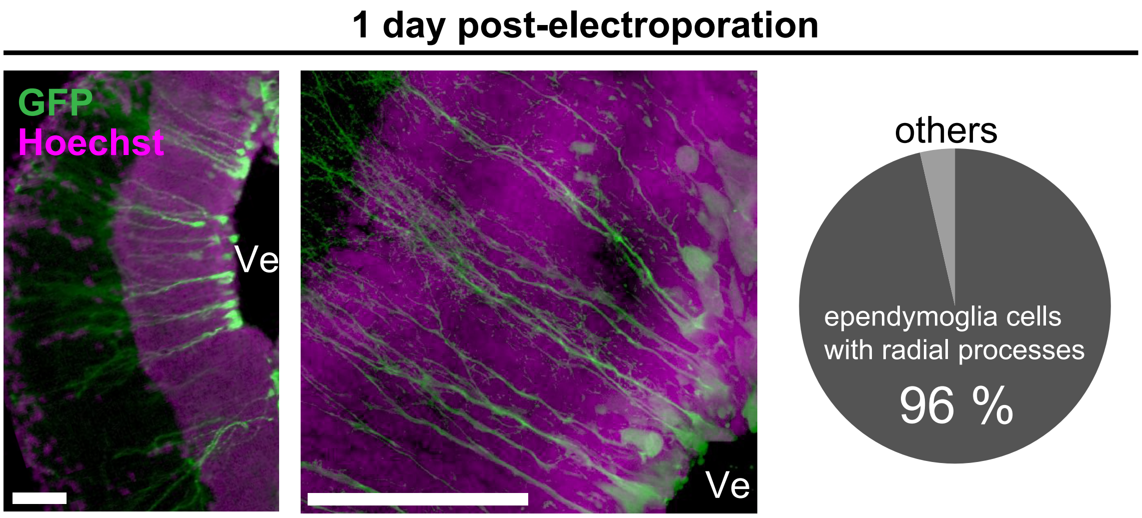

Fig. S6 (related to Fig. 6). Labeling of ependymoglia cells by in vivo electroporation.

Immunohistochemistry for GFP on coronal sections of the newt mesencephalon (n=4 animals, n=4 sections) at 1 day after electroporation, and efficiency of gene transfer into ependymoglia cells. The majority (96%) of first labeled cells were ventricular ependymoglia cells possessing long radial processes. Scale bars: 100 μm.

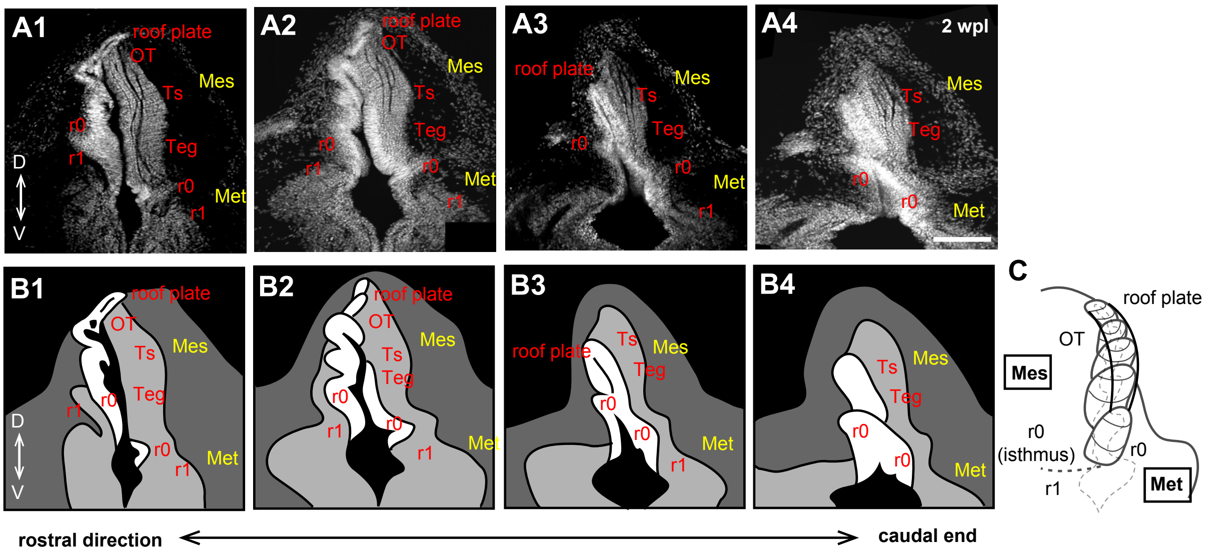

Fig. S7 (related to Fig. 6). Tissue architecture of the caudal pole of the regenerating mesencephalon.

(A1-A4) Hoechst staining of serial coronal sections of the caudal pole of the regenerating mesencephalon at 2 wpl, showing that the thickened ependymal layer forms a segmental curved shape. (B1-B4) Schematic representation of section levels indicated in A1-A4. The thickened neuroepithelial-like ependymal layer (white), the neuronal cell layer and typical ependymal layer (light gray), the axonal layer (dark gray) are depicted. (C) Schematic representation of pseudo-3D reconstruction from multiple images of B1-B4, showing the segmental curved shape of the regenerating ependymal layer in the caudal pole of the mesencephalon. Scale bars: 250 µm.

Biology Open • Supplementary information
