# Supplementary material for: Spatio-temporal neural stem cell behavior leads to both perfect and imperfect structural brain regeneration in adult newts
Source: Biol Open. 2018 Jun 14;7(6):bio033142. doi: 10.1242/bio.033142 (PMC6031346; doi:10.1242/bio.033142)
Supplement: First Person interview [file biolopen-7-033142-s2.pdf]

© 2018. Published by The Company of Biologists Ltd | Biology Open (2018): doi:10.1242/bio.033142: Supplementary information

FIRST PERSON

First person – Yuko Urata

First Person is a series of interviews with the first authors of a selection of papers published in Biology Open, helping early-career researchers promote themselves alongside their papers. Yuko Urata is first author on 'Spatio-temporal neural stem cell behavior leads to both perfect and imperfect structural brain regeneration in adult newts', published in BiO. Yuko is a PhD student in the lab of Kiyokazu Agata at Kyoto University, Kyoto, Japan, seeking to capture biological phenomena through comparison of relative scales, rather than through absolute scales such as gene function in specific organisms.

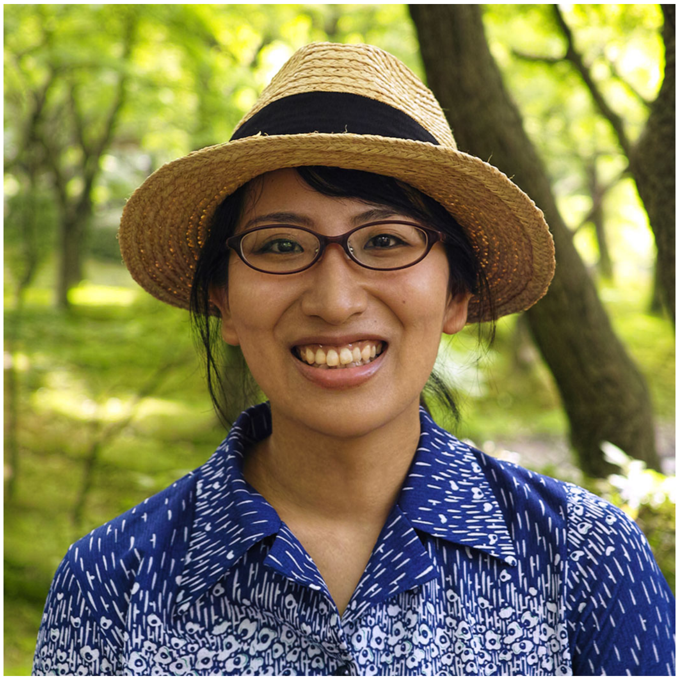

Yuko Urata

What is your scientific background and the general focus of your lab?

When I was an undergraduate student of biology, I was fascinated by mathematics and physics, and loved to think about how to understand complicated systems. I wanted to continue to investigate systems from a higher perspective after resolving as many different aspects as possible in time and space, so I chose the laboratory of developmental biology and evolution. During my undergraduate studies, I started studying genome evolution with duplicated silkworm genes under Prof. Masanobu Ito at the Kyoto Institute of Technology. I then moved to Prof. Kiyokazu Agata's laboratory at Kyoto University to study regeneration using newts (sometimes *Xenopus*) for my master's and PhD degrees. Planarians are the main experimental animals in the Agata lab; however, there were various other organisms including freshwater demosponges, dark flies, polka-dotted fruit flies, newts, *Xenopus*, chick embryos, geckos, mice, cultured cell lines and primate cells, and I find inspiration in all of these. There is a common theme across regeneration groups on 'learning the principle of regeneration from organisms that can regenerate', and also an atmosphere of doing our best to study newt regeneration under the influence of Prof. Agata's mentor, the late Dr Tokindo S. Okada.

How would you explain the main findings of your paper to non-scientific family and friends?

First of all, everyone is surprised that adult newts can regenerate large portions of the brain. In contrast to mammals, including humans, adult newts can regenerate various parts of the body such as limbs, lenses, heart, brain etc. from cells called 'adult stem cells', producing various kinds of differentiated cells. However, it is difficult to imagine how to reconstruct complex organ structures from stem cells. We used brain regeneration as a model for structural regeneration because in the newt brain, neural stem cells neatly line the ventricle walls as the brain develops, and we have tools available to define brain sub-regions at the cellular level. This allows us to address what kind of stem cell behavior is related to the reconstruction of complicated structures. In our paper, we follow the long-term regeneration process after large-scale brain excision, and show that stem cells proliferate and differentiate in time and space. We discovered that there are both perfect and imperfect parts of the regenerated structure within an individual's brain. By comparing both regeneration processes, we found that perfect regeneration is accomplished by the coordinated behavior of stem cells around specific brain regions to form a stem cell sheet in the ventricle walls, with developmental processes resembling those observed in embryonic development. By contrast, the imperfect structure originates from an abnormal gap in this sheet. In short, certain conditions must be met in order to properly regenerate the brain structure, even in newts with high regenerative capacity.

Yuko Urata's contact details: Department of Biophysics, Graduate School of Science, Kyoto University, Kitashirakawa-Oiwake, Sakyo-ku, 606-8502 Kyoto, Japan.
E-mail: urata.yuko.73s@kyoto-u.jp

What are the potential implications of these results for your field of research?

Our research is composed of descriptive works, and sometimes people think descriptive results are not sophisticated because they lack mechanistic insights. However, regeneration is an inherently complicated process with a combination of many internal and external conditions and factors. Thus, in some cases, you notice what you should pay attention to the first time by carefully describing processes to get a bird's-eye view. Now that we have discovered some analogies between brain regeneration and development, I expect that from an evo-devo perspective, it will be possible to investigate reasons why vertebrates have different regenerative abilities. Also, although the path to translational studies is never a straight line, I hope this will help.

What has surprised you the most while conducting your research?

I spent a long time observing histological staining and immunostaining results without obtaining a panoramic view of

Biology Open (2018): doi:10.1242/bio.033142: Supplementary information

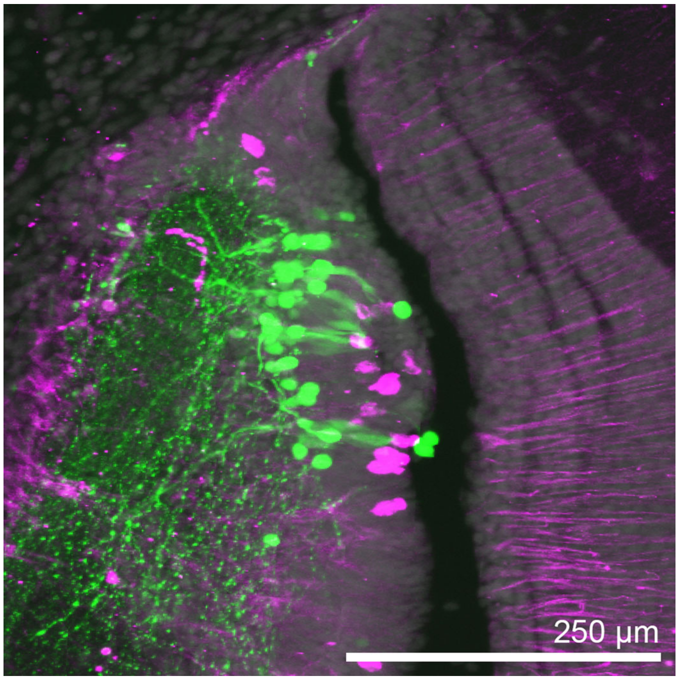

A regenerating newt brain at 2 weeks post-lesion (nuclei, gray). Stem cells lining the ventricular surface (their radial processes and mitotic phase, magenta), which was initially labeled by electroporation, produce progeny (green) to lesioned regions.

brain regeneration. I was gradually able to get reproducible results, and the moment the scattered points connected was one of the most pleasant surprises for me. In addition, the many links we made between our results and previous studies was another pleasant surprise for me. I am always inspired by other people and would like to say thank you to the authors of these other studies for their involvement.

> "[…] the many links we made between our results and previous studies was another pleasant surprise for me."

What, in your opinion, are some of the greatest achievements in your field and how has this influenced your research?

I am fascinated by the explosion in organoid studies. I think that my research has been very heavily influenced by the concept of modularity and self-organization of organs rather than technical aspects of organoid studies. Though these concepts have been around for a long time, it has become possible to approach various questions experimentally by combining organoids with CRISPR/Cas9 and other systems.

What changes do you think could improve the professional lives of early-career scientists?

I think that academic communities should eliminate the current trend that early-career scientists must never fail or stop even once. There have been a number of aspects of my research that did not take shape, but I believe that each of these led to my self-improvement. In Japan, most early-career scientists are in a highly unstable financial position and it becomes a situation where researchers cannot keep studying unless their success continues to run in a straight line. This situation will also reduce the number of scientists with diverse backgrounds. A longer-term perspective needs to be considered, along with promoting a more productive environment.

What's next for you?

Completing my PhD. Developing new techniques and continuing to thoughtfully address fascinating questions wherever I am.

Reference

Urata, Y., Yamashita, W., Inoue, T. and Agata, K. (2018). Spatio-temporal neural stem cell behavior leads to both perfect and imperfect structural brain regeneration in adult newts. *Biol. Open* **7**: bio033142.

Biology Open
